# Characterization of Novel PI3Kδ Inhibitors as Potential Therapeutics for SLE and Lupus Nephritis in Pre-Clinical Studies

**DOI:** 10.3389/fimmu.2014.00233

**Published:** 2014-05-22

**Authors:** Philipp Haselmayer, Montserrat Camps, Mathilde Muzerelle, Samer El Bawab, Caroline Waltzinger, Lisa Bruns, Nada Abla, Mark A. Polokoff, Carole Jond-Necand, Marilène Gaudet, Audrey Benoit, Dominique Bertschy Meier, Catherine Martin, Denise Gretener, Maria Stella Lombardi, Roland Grenningloh, Christoph Ladel, Jørgen Søberg Petersen, Pascale Gaillard, Hong Ji

**Affiliations:** ^1^Immunology, Department of Preclinical Pharmacology, Merck Serono, Darmstadt, Germany; ^2^Biologics and Immunology Platform, Merck Serono, Darmstadt, Germany; ^3^Department of Cellular Immunology, Merck Serono SA, Geneva, Switzerland; ^4^Biologics and Immunology Platform, Merck Serono SA, Geneva, Switzerland; ^5^Department of Chemistry, Merck Serono SA, Geneva, Switzerland; ^6^Drug Metabolism and Pharmacokinetics (DMPK), Non-Clinical Development, Merck Serono, Darmstadt, Germany; ^7^Drug Metabolism and Pharmacokinetics (DMPK), Non-Clinical Development, Merck Serono SA, Geneva, Switzerland; ^8^BioSeek^^®^^ Division, DiscoveRx Corporation, South San Francisco, CA, USA; ^9^Department of Early PK/PD Biomarker, Merck Serono SA, Geneva, Switzerland; ^10^Department of Screening, Merck Serono SA, Geneva, Switzerland; ^11^Immunology, Department of Preclinical Pharmacology, EMD Serono Research and Development Institute, Billerica, MA, USA

**Keywords:** PI3Kδ inhibitor, immune response, SLE, lupus nephritis, drug development, pharmacodynamic biomarker, pharmacokinetic/pharmacodynamic modeling

## Abstract

SLE is a complex autoimmune inflammatory disease characterized by pathogenic autoantibody production as a consequence of uncontrolled T–B cell activity and immune-complex deposition in various organs, including kidney, leading to tissue damage and function loss. There is a high unmet need for better treatment options other than corticosteroids and immunosuppressants. Phosphoinositol-3 kinase δ (PI3Kδ) is a promising target in this respect as it is essential in mediating B- and T-cell function in mouse and human. We report the identification of selective PI3Kδ inhibitors that blocked B-, T-, and plasmacytoid dendritic cell activities in human peripheral blood and in primary cell co-cultures (BioMAP^®^) without detecting signs of undesired toxicity. In an IFNα-accelerated mouse SLE model, our PI3Kδ inhibitors blocked nephritis development, whether administered at the onset of autoantibody appearance or the onset of proteinuria. Disease amelioration correlated with normalized immune cell numbers in the spleen, reduced immune-complex deposition as well as reduced inflammation, fibrosis, and tissue damage in the kidney. Improvements were similar to those achieved with a frequently prescribed drug for lupus nephritis, the potent immunosuppressant mycophenolate mofetil. Finally, we established a pharmacodynamics/pharmacokinetic/efficacy model that revealed that a sustained PI3Kδ inhibition of 50% is sufficient to achieve full efficacy in our disease model. These data demonstrate the therapeutic potential of PI3Kδ inhibitors in SLE and lupus nephritis.

## Introduction

SLE is a complex autoimmune inflammatory disease that affects multiple organs with unpredictable flares (1). It is characterized by pathogenic autoantibodies, immune-complex deposition in target tissues, and ensuing inflammation and organ damage. Although the etiology of SLE is poorly understood, genetic and environmental factors play an important role and contribute to the deregulation of both innate and adaptive immune systems. A hallmark is the breakdown of B- and/or T-cell self-tolerance and the appearance of autoantibodies directed against nuclear components, such as anti-dsDNA antibodies. Autoantibody/antigen immune-complexes in turn can activate TLR7 and/or TLR9 in plasmacytoid dendritic cells (pDCs) leading to the production of IFNα. IFNα stimulates myeloid DC maturation that promotes loss of self-tolerance, autoantibody production, immune-complex formation, and further production of IFNα, sustaining a self-perpetuating vicious cycle of autoimmunity in SLE. Thirty to fifty percent of SLE patients develop lupus nephritis that can lead to renal failure, a major life-threatening complication. Commonly used therapies include corticosteroids, immunosuppressants/cytotoxic drugs [such as mycophenolate mofetil (MMF)], and hydroxychloroquine. These drugs block B-, T-cell, or TLR9 function and improve SLE symptoms, but their long-term use is associated with moderate to severe side effects. The development of novel therapeutics to treat SLE and lupus nephritis has been challenging due to the complex nature of the disease and the heterogeneity of clinical manifestation (2). In over 50 years, belimumab, a human monoclonal Ab that neutralizes B cell-activating factor (BAFF) was the first new drug approved for the treatment of SLE (3). By interfering with B cell differentiation, belimumab is believed to prevent autoreactive B cells from becoming Ab-producing plasma cells thereby decreasing SLE autoantibody titers. Belimumab has a favorable safety profile but shows only modest efficacy in SLE and is not approved for lupus nephritis, suggesting that blocking B cell function alone is not sufficient to curb the disease. For SLE therapeutics to be highly effective, it appears that not only B cells but also other key pathogenic components, such as T-cells and possibly pDC, TLR, and/or IFNα activities need to be controlled – without compromising long-term safety.

Class I phosphoinositide 3 kinases (PI3Ks), comprising α, β, γ, and δ isoforms, are fundamental components of cell signaling as they generate phosphatidylinositol (3,4,5)-triphosphate at the plasma membrane that recruits and activates proteins containing phospholipid-binding domains, such as Akt, to initiate downstream signaling events leading to cell growth, survival, differentiation, proliferation, migration, or cytokine release (4). PI3Kδ together with PI3Kα and PI3Kβ isoforms belong to class IA PI3Ks, which are activated by tyrosine kinase receptors, whereas PI3Kγ, the only member of Class IB PI3K family, mediates signaling downstream of G-protein-coupled receptors. Unlike PI3Kα and PI3Kβ, which are ubiquitously expressed, PI3Kδ and PI3Kγ isoforms show a restricted expression and are found mainly in hematopoietic and endothelial cells. While PI3Kγ plays major roles in myeloid cell function (5), PI3Kδ is pivotal in mediating B cell receptor (BCR)- and T-cell receptor (TCR)-mediated responses, including B cell proliferation, differentiation, homing, Ag presentation, Ab and cytokine production, T-cell differentiation and cytokine production (6), and TLR9-induced IFNα production in pDC (7). All of these activities are considered major pathogenic contributors in SLE. In addition, PI3Kδ activity confers resistance to activation-induced T-cell death (AICD), a defect that may contribute to loss of self-tolerance in SLE: two-thirds of studied patients show enhanced PI3Kδ activation in correlation with disease activity (8). Indeed, haploinsufficiency of PI3Kδ in mice leads to defects in T-cell activation, Ab class-switching, and decreased SLE pathology in Lyn-deficient mice (9). Thus, specific inhibition of PI3Kδ may provide the appropriate immunomodulation to control SLE pathology.

Here, we describe the discovery and characterization of two potent and selective small molecule PI3Kδ inhibitors, MSC2360844 and MSC2364588. We show that both compounds were potent inhibitors of PI3Kδ-mediated cell functions of B- and T-cells, and to a lesser extent pDCs in human primary cell cultures. In an IFNα-accelerated NZB/W F1 SLE model, MSC2360844 and MSC2364588 blocked the development of proteinuria and kidney damage in a dose-dependent manner. In addition, we established a pharmacokinetic (PK − compound exposure)/pharmacodynamic (PD − compound effect)/efficacy model. Using this model, we were able to determine the percentage of required sustained PI3Kδ inhibition for optimal efficacy. The model and data obtained will facilitate potential clinical development.

## Materials and Methods

### Chemical synthesis

MSC2360844, 6-fluoro-3-(morpholin-4-yl carbonyl)-1-[4- (morpholin-4-yl methyl) phenyl]-1,4-dihydrothiochromeno[4,3-c]pyrazole 5,5-dioxide, was synthesized in five steps following procedures described in WO2011058149. As a summary, synthesis was started by reaction of 6-fluoro-thiochroman-4-one with diethyl oxalate in the presence of sodium ethoxide. The intermediate was then cyclized with 4-(4-hydrazinylbenzyl)morpholine forming a pyrazole ring. The thioether was oxidized to the corresponding sulfone by reaction with meta-chloroperbenzoic acid which was followed by the saponification of the ethyl ester into the corresponding acid followed by the coupling with morpholine to give the desired compound.

MSC2364588, (R)-7-fluoro-3-(morpholin-4-yl carbonyl)-1-[1-(2-morpholin-4-yl ethyl)piperidin-3-yl]-1,4-dihydrothiochro- meno[4,3-c]pyrazole 5,5-dioxide, was synthesized in nine linear steps as described in WO2011058149. As a summary, synthesis was started by reaction of 7-fluoro-thiochroman-4-one with diethyl oxalate in the presence of sodium ethoxide. The intermediate was then cyclized, in the presence of HCl, with tert-butyl 3-[2-(tert-butoxycarbonyl)hydrazino]piperidine-1-carboxylate, that was obtained by reduction of the hydrazone resulting from the reaction of 1-boc-3-piperidone with tert-butyl-carbazate, forming a pyrazole ring. The piperidine ring was protected by reaction with di-tert-butyl dicarbonate and the thioether was oxidized to the corresponding sulfone by reaction with meta-chloroperbenzoic acid. The intermediate was then deprotected in the presence of TFA, separated by chiral chromatography, and (R)-7-fluoro-3-(morpholin-4-yl carbonyl)-1-[piperidin-3-yl]-1,4-dihydrothiochromeno[4,3-c]pyrazole 5,5-dioxide was reacted with chloroacetaldehyde and sodium triacetoxyborohydride. The addition of morpholine in the presence of sodium iodide and potassium carbonate yielded the desired compound.

### Compounds

MSC2360844 and MSC2364588 were dissolved in DMSO for *in vitro* studies and in 0.5% hydroxypropyl methylcellulose (HPMC) and 0.25% (v/v) Tween 20 in water for *in vivo* studies by gavage at indicated concentrations or doses.

### Biochemical assay

Scintillation proximity assay (SPA) was performed to assess PI3Kα, β, γ, and δ enzymatic activity using neomycin-coated beads (Amersham, Buckinghamshire, UK) with 100 μM of phosphatidyl inositol in form of lipid micelles as substrates containing phosphatidyl-l-serine (Sigma) as lipid carrier and 65 μM [γ33P]ATP (10). The scintillation signal was measured in a 384 plate reader, Triluxβ-counter (Perking Elmer, Shelton, CT, USA).

### Compound profiling in BioMAP^®^ systems

Protocols for compound profiling in complex primary human cell culture (BioMAP) systems have been reported previously (11–13), and are described in detail in Section 3 in Supplementary Material. Preparation and culture of primary human endothelial cells, neonatal foreskin fibroblasts (HDFn) and bronchial epithelial cells (Cell Applications, Inc., San Diego, CA, USA), arterial smooth muscle cells and keratinocytes (Lonza, Inc., Allendale, NJ, USA) were as previously described. Positively selected primary normal human CD19^+^ B cells and CD4^+^ T-cells were obtained from AllCells (Emeryville, CA, USA). PBMC were isolated from buffy coats (Stanford Blood Center, Stanford, CA, USA). All primary human cells utilized in this work were obtained under protocols that were reviewed by Institutional Review Board(s) (IRB) that operate in accordance with the requirement of EPA Regulation 40 CFR 26 and HHS Regulation 45 CFR 46 of the US Federal Government for the protection of human research subjects.

Test compounds were prepared in DMSO, added 1 h before stimulation of the cells, and were present during the whole 24–144 h stimulation period, depending on system.

The levels of readout parameters were measured by ELISA (14, 15). Measurement values for each biomarker readout in a compound-treated sample were divided by the mean value from eight DMSO control samples (from the same plate) to generate a ratio. All ratios were then log10 transformed and plotted (BioMAP profile). Overt cytotoxicity of test compounds was assessed by total cell protein staining sulforhodamine B (Sigma-Aldrich, St. Louis, MO, USA) for adherent cells, or by cell viability staining Alamar Blue (Invitrogen, Carlsbad, CA, USA) for non-adherent cells.

### Human sample

Heparinized human blood or buffy-coat prepared from healthy donors was obtained from the “Center de transfusion sanguine” of Geneva (Switzerland). Cryopreserved PBMCs from SLE patients were obtained from Asterand (Detroit, MI, USA). Both SLE patients were female with moderate clinical activity (SLEDAI score of 6 at the day of blood draw) and treated with hydroxychloroquine and NSAIDs. The patients were 55 and 65 years old. Studies were approved by Merck Serono ethics committee.

### BCR-crosslinking-induced Akt phosphorylation in cellular assays

Human B lymphocyte cell line Ramos cells (ATCC #CRL-1923) were grown in serum-free IMDM medium for 2 h, incubated with PI3Kδ inhibitors for 20 min, and stimulated with 10 μg/ml anti-IgM (Jackson Immuno Research, West Grove, PA, USA) for 15 min. Cells were fixed with 4% paraformaldehyde and permeabilized in 0.2% Triton X-100 in PBS before staining with rabbit anti-phospho-Akt (pAkt) (Ser473) (Cell Signaling Technology, Danvers, MA, USA) in PBS containing 4% FCS for 1 h, followed by mouse anti-human IgM–APC (BD Biosciences Pharmingen, San Diego, CA, USA) and anti-rabbit IgG-Alexa 488 (Invitrogen, Carlsbad, CA, USA) for 30 min at room temperature.

#### Anti-IgM-induced pAkt in human whole blood

Human whole blood was incubated with PI3Kδ inhibitors for 30 min and stimulated with 30 μg/ml anti-IgM (Jackson Immuno Research, West Grove, PA, USA) for 5 min at 37°C. Samples were fixed with 4% formaldehyde for 8 min and red blood cells were lysed with 0.16% Triton for 30 min at 37°C followed by permeabilization with 50% methanol overnight at −20°C. Cell were stained with rabbit anti-pAkt (Ser473) followed by the Alexa647-conjugated F(ab′)2 goat anti-rabbit IgG (Invitrogen, Carlsbad, CA, USA), and anti-CD19-PE (BD Biosciences Pharmingen, San Diego, CA, USA). CD19^+^ cells were gated for pAkt analysis.

#### Anti-IgD-induced pAkt in mouse whole blood

Whole blood withdrawn from C57BL/6N mice (Charles River Laboratories, L’Arbresle, France) was incubated with PI3Kδ inhibitors for 30 min and stimulated with goat anti-mouse IgD antiserum (eBioscience, San Diego, CA, USA) for 7 min at 37°C. Samples were fixed with 4% formaldehyde for 6 min and red blood cells were lysed with 0.16% Triton for 30 min at 37°C followed by permeabilization with 50% methanol overnight at −20°C. Cells were stained with rabbit anti-pAkt (Ser473) for 1 h followed by anti-IgD-FITC, anti-B220-PerCP-Cy5.5 (all BD Biosciences Pharmingen, San Diego, CA, USA), and donkey anti-rabbit IgG-Alexa647 (Invitrogen, Carlsbad, CA, USA) for 30 min at room temperature. B220^+^IgD^+^ cells were gated for pAkt analysis.

### BCR-crosslinking-induced CD69 up-regulation in whole blood assays

#### Anti-IgM-induced CD69 in human whole blood

Human whole blood were incubated with PI3Kδ inhibitors for 30 min and stimulated with 30 μg/ml anti-IgM for 18 h before staining with anti-CD69-PE and anti-CD19-PerCp-Cy5.5 (all BD Biosciences Pharmingen, San Diego, CA, USA) for 30 min at room temperature. After red blood cells lysis, CD19^+^ cells were gated for CD69 analysis.

#### Anti-IgM-induced CD69 in mouse whole blood

Whole blood withdrawn from naïve C57BL/6N mice (Charles River Laboratories, L’Arbresle, France) were incubated with PI3Kδ inhibitors for 30 min and stimulated with 1/12 goat anti-mouse IgD antiserum (eBioscience, San Diego, CA, USA) for 4 h before staining with anti-B220-PerCP-Cy5.5 and anti-CD69-PE (all BD Biosciences Pharmingen, San Diego, CA, USA) for 30 min at room temperature. After red blood cells lysis, B220^+^IgD^+^ cells were gated for CD69 analysis.

### *Ex vivo* mouse whole blood assays

PI3Kδ inhibitors were administered to naïve C57BL/6N mice (Charles River Laboratories, L’Arbresle, France), by gavage at indicated time points before heparinized whole blood was obtained and divided into two aliquots. One aliquot was incubated with anti-IgD as stimulation and another PBS as basal control. pAkt in B cells or CD69 up-regulation on B cell surface of each sample was measured as described for the *in vitro* assays. The difference between stimulated and basal levels of mean fluorescence intensity (MFI) was calculated as Δ value.

### Proliferation assay

CD19^+^ B cells were isolated from PBMC of healthy volunteers by negative selection using the B cell purification kit II (Miltenyi Biotech, Bergisch Gladbach, Germany) and following manufacturer’s instructions. Purified B cells (with a purity of more than 95%) were incubated with PI3Kδ inhibitors for 1 h, and stimulated with 10 μg/ml goat F(ab′)2 anti-IgM (Southern Biotech) and 10 ng/ml recombinant human IL-4 (Immunotools, Friesoythe, Germany) for 4 days. [^3^H] thymidine (1 μCi; Perkin Elmer, Shelton, CT, USA) was added for the last 18 h of culture. Proliferation was assessed by a multiplate beta counter (Perkin Elmer, Shelton, CT, USA).

### Cytokine release assays

#### B cell cytokine release assay

CD19^+^ B cells isolated PBMC of healthy volunteers (with a purity of more than 95%) were incubated with PI3Kδ inhibitors for 1 h, and stimulated 10 μg/ml rabbit anti-human IgA + IgG + IgM (H + L) (Jackson Laboratories, Bar Harbor, ME, USA), 3 μg/ml CpG ODN 2006 (oligodeoxynucleotides; InVivogen, San Diego, CA, USA) and 8000 IU/ml of recombinant human IFN-α for 48 h. Cytokines in the supernatants were measured with Cytometric Bead Array kits (BD Biosciences Pharmingen, San Diego, CA, USA).

#### T-cell cytokine release assay

CD4^+^CD45RA^−^ memory T-cells (more than 95% purity) were isolated from PBMC of healthy volunteers by negative selection using the CD4^+^ memory cell purification kit (Miltenyi Biotech, Bergisch Gladbach, Germany). Purified T-cells were incubated with PI3Kδ inhibitors for 1 h, and stimulated with 5 μg/ml anti-CD3 and 2 μg/ml anti-CD28 for 5 days. Cytokines in the supernatants were measured with Luminex^®^ xMAP^®^ multiplex bead assay kits (Bio-Rad, Hercules, CA, USA).

#### Plasmacytoid dendritic cells IFN-α release assay

Plasmacytoid dendritic cells were isolated from PBMC of healthy volunteers by negative selection using pDC purification kit (Miltenyi Biotech, Bergisch Gladbach, Germany). Purified CD123^+^/CD303^+^ pDC cells (with more than 95% of purity) were incubated with PI3Kδ inhibitors for 1 h and stimulated with 1 μM CpG ODN 2395 Type C (InVivogen, San Diego, CA, USA) for 18 h. IFN-α in the supernatants was measured from the culture supernatants with Verikine ELISA kit (PBL Assay Science, Piscataway, NJ, USA).

### PK/PD modeling

An *I*_max_ PK/PD model describing BCR activation-induced pAkt response was established using data from *in vitro* and *ex vivo* mouse whole blood assays.

$$E=E_{0}-I_{\text{max}}\times C∕\left(\text{I}{\text{C}}_{50}+C\right),$$

where *E* represents pAkt response, *C* compound plasma concentration, *E*_0_ pAkt upon *ex vivo* stimulation in the absence of compound treatment, *I*_max_ the maximum inhibitory effect, and IC_50_, concentration required to achieve 50% of *I*_max_ *in vitro*.

### Accelerated NZB/W F1 mouse SLE model

All protocols and procedures were conducted according to German animal protection law (Tierschutzgesetz) and were approved by the local veterinarian authorities (Regierungspräsidium, Darmstadt, Germany). Ten-week-old female NZB/W F1 mice (Jackson Laboratories, Bar Harbor, ME, USA) were given a single i.v. injection of 1 × 10^8^ IU/100 μl of AdV-IFN-α (adenovirus with mmIfna5_v1 insert, Biofocus, CoA.NL.09.160) in saline or left untreated (sham). Drug treatments were either initiated at 2 weeks (early intervention regimen) or 4 weeks (late intervention regimen) post AdV-IFN-α delivery until the end of the experiment (at 10 weeks). Mice (10 per group) were treated once or twice daily with MSC2360844 at indicated doses or MMF (Cell Cept, Roche) at 300 mg/kg once daily in NaCl by gavage, or BAFF-R-Ig (Merck Serono) at 5 mg/kg, or mouse Fc (mFc, Merck Serono) at 5 mg/kg as isotype control in PBS by intraperitoneal injection. Serum and urine samples were collected for anti-dsDNA antibodies (by ELISA) and urinary protein creatinine ratio (UPCR) (measured by ADVIA 1800) determination, respectively, on the days indicated. Proteinuria is defined as UPCR >3.

#### Histopathology analysis of kidney

Paraffin-embedded H&E-stained 4 μm tissue sections were blindly scored as the sum of the following three systems including inflammation, glomerular damage, and sclerosis. Inflammation: Grade 1, mild focal interstitial inflammation; Grade 2, multifocal areas of mild interstitial inflammation; Grade 3, moderate multifocal interstitial inflammation; Grade 4, significant multifocal interstitial inflammation. Glomerular damage: Grade 1, initial glomerular lesions; initial lesions are characterized by increased cellular components in a single to few glomeruli with cellular proliferation and thickening of basement membrane (membranoproliferative/mesangiocapillary); Grade 2, multifocal areas of glomerular lesions; Grade 3, multifocal areas of glomerular lesions with significant damage characterized by proliferation of epithelial cells of capsule of Bowman with compression of glomerular capillaries; Grade 4, pronounced multifocal to generalized glomerular damage (i.e., Grade 3 plus obliteration of Bowman space, glomerular compression, and hyalinosis). Glomerular sclerosis: Grade 1, focal mild glomerular sclerosis; Grade 2, multifocal mild glomerular sclerosis; Grade 3, severe focal glomerular sclerosis; Grade 4, severe multifocal glomerular sclerosis.

#### Detection of antibody-secreting cells

ELISpot plates (Merck Millipore, Billerica, MA, USA) were coated with 10 μg/ml goat anti-mouse IgG (Calbiochem). Spleen cells were harvested and titrated starting with 300,000 cells/well. Cells were incubated for 5 h and after washing and incubation with secondary HRP conjugated anti-mouse IgG (Sigma, St. Louis, MO, USA). ELISpots were developed with AEC staining Kit (Sigma, St. Louis, MO, USA). ELISpots were counted using ELISPOT reader (AID, Strassberg, Germany).

#### Determination of anti-dsDNA level

ELISA plates were coated with 10 μg thymic DNA (Sigma, St. Louis, MO, USA). Serum of each animal was titrated to determine anti-dsDNA antibody levels. The levels of each individual were calculated as arbitrary units compared to a pooled standard serum from 22-week-old MRLlpr/lpr mice. Detection was performed using goat anti-mouse IgG HRP-coupled antibodies and 3,3′,5,5′-tetramethylbenzidine (Sigma-Aldrich, St. Louis, MO, USA).

#### Immunofluorescence staining

Cryosections (5 μm) of kidneys were stained for 1 h at room temperature with FITC-conjugated anti-mouse IgG2a (Southern Biotech, Birmingham, AL, USA). Images were captured using a CCD camera (AXIOCamMRm Zeiss, Berlin, Germany) connected to a Zeiss microscope.

## Results

### Identification of novel PI3Kδ-specific small molecule inhibitors

**Figure 1:**
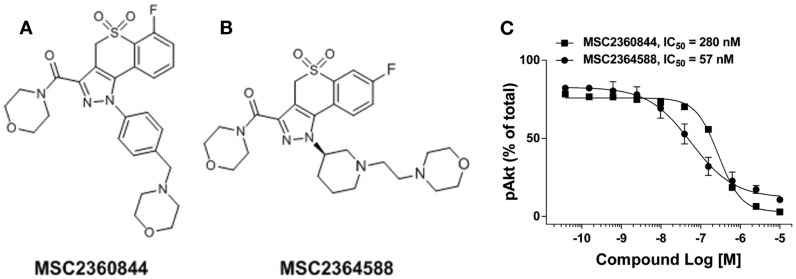
**Identification of PI3Kδ inhibitor MSC2360844 and MSC2364588**. **(A)** Chemical structure of MSC2360844. **(B)** Chemical structure of MSC2364588. **(C)** Ramos cells were stimulated with anti-IgM for 15 min. Intracellular pAkt was measured by flow cytometry. Data shown are mean ± SEM and are representative of three experiments.

We identified a thiochroman series through screening of a focused kinase inhibitor compound library (60 K) for the inhibition of PI3Kδ activity. Compounds were selected from both, commercial and proprietary sources, based on in-house knowledge of the target. Further hit to lead optimization efforts resulted in the design and synthesis of MSC2360844 (6-fluoro-3-(morpholin-4-yl carbonyl)-1-[4-(morpholin-4-ylmethyl)phenyl]-1,4-dihydrothiochromeno[4,3-c] pyrazole 5,5-dioxide) (Figure 1A) and MSC2364588 (7-fluoro-3-(morpholin-4-yl carbonyl)-1-[(3R)-1-(2-morpholin-4-yl ethyl)piperidin-3-yl]-1,4-dihydrothiochromeno[4,3-c]pyrazole 5,5-dioxide) (Figure 1B). MSC2360844 and MSC2364588 inhibited PI3Kδ enzymatic activity with IC_50_ values of 145 and 15 nM, respectively, whereas PI3Kα, PI3Kβ, and PI3Kγ were inhibited only in the micromolar range (Table 1).

**Table 1:** **Enzymatic potency of MSC2360844 and MSC2364588 in human class I PI3K isoforms**.

Enzymatic potency IC_50_ (nM)		
Isoform	MSC2360844	MSC2364588
PI3K δ	145	15
PI3K α	18,500	7,650
PI3K β	2,850	2,450
PI3K γ	>20,000	>20,000

MSC2360844 and MSC2364588 were also highly selective against a panel of 278 additional kinases (Figure S1 in Supplementary Material). At 10 μM, MSC230844 did not show significant inhibition (>50%) of any kinase, while MSC2364588 impaired Hck and PAK3 activity albeit with IC_50_ > 10 μM. In addition, both compounds showed favorable *in vitro* and *in vivo* drug metabolism and pharmacokinetic (DMPK) properties (Tables S1 and S2 in Supplementary Material), leading us to progress MSC2360844 and MSC2364588 to further pharmacological characterization.

PI3Kδ is the only PI3K isoform that mediates in B cells Ag receptor-induced Akt phosphorylation (pAkt), a surrogate marker for PI3K cellular activation (16). MSC2360844 and MSC2364588 completely abolished BCR-induced pAkt in Ramos B cells in a concentration-dependent manner with IC_50_ values of 280 and 57 nM, respectively (Figure 1C). These values correlate with their relative enzymatic potency (Table 1) and cell permeability (Tables S1 and S2 in Supplementary Material).

Thus, MSC2360844 and MSC2364588 are effective and selective PI3Kδ inhibitors in both enzymatic and cellular contexts, with the latter compound showing 10-fold higher potency.

### MSC2360844 and MSC2364588 inhibit SLE-prone functions of human primary cells

**Figure 2:**
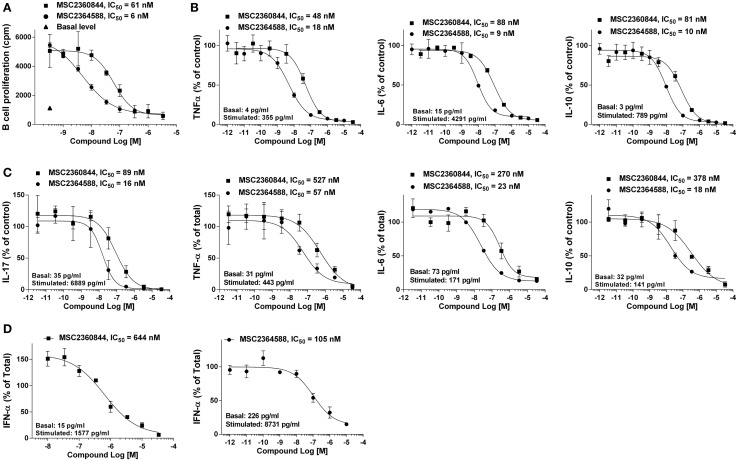
**MSC2360844 and MSC2364588 block BCR- and TCR-mediated responses in lymphocytes and TLR-induced IFNα by pDC in human primary cells**. **(A)** CD19^+^ B cells isolated from PBMCs of healthy donors were stimulated with anti-IgM and IL-4 for 4 days. Proliferation was measured by [^3^H]-thymidine incorporation. **(B)** CD19^+^ B cells isolated from PBMCs of healthy donors were stimulated with anti-Ig, CpG ODN 2006, and IFNα for 48 h. Cytokines in the supernatants were measured with CBA kits. **(C)** CD4^+^CD45RA^−^ T-cells isolated from PBMCs of healthy donors were stimulated with anti-CD3 and anti-CD28 for 5 days. Cytokines in the supernatants were measured with Luminex kit. **(D)** CD123^+^/CD303^+^ pDC cells isolated from PBMCs of healthy donors were stimulated with CpG ODN 2395 Type C for 18 h. IFNα in the supernatants were measured with ELISA kit. Difference between basal and stimulated level was used as 100%. Data shown are mean ± SEM and are representative of at least three independent experiments.

We assessed the effects of MSC2360844 and MSC2364588 on B- and T-cells and pDCs, the key contributors to SLE pathogenesis. To this end, we isolated CD19^+^ B cells from PBMC of healthy donors and stimulated them *in vitro* with F(ab′)2 anti-IgM and IL-4 for 4 days to induce B cell proliferation. MSC2360844 and MSC2364588 inhibited B cell proliferation in a concentration-dependent manner with an IC_50_ of 48 and 7 nM, respectively (Figure 2A). To simulate pro-inflammatory conditions observed in SLE patients, we cultured B cells in a cocktail containing F(ab′)2 anti-IgM, CpG, and IFNα for 2 days and measured cytokine levels in the supernatants. MSC2360844 and MSC2364588 reduced TNFα, IL-6, and IL-10 with potencies similar to those observed in the B cell proliferation assay (Figure 2B). Thus, both MSC2360844 and MSC2364588 block B cell function effectively. MSC2364588 was again 10 times more potent as seen before in direct PI3Kδ enzymatic inhibition.

In SLE, T-cell cytokines such as IL-17, IL-6, TNFα, and IL-10 are up-regulated and their abundance correlates with disease activities (17). Both of our compounds inhibited these cytokines produced by CD4^+^CD45RA^−^ memory T-cells from healthy donors upon anti-CD3 and anti-CD28 stimulation for 5 days in a concentration-dependent manner, again with MSC2364588 being 10 times more potent than MSC2360844 (Figure 2C). Interestingly, both PI3Kδ inhibitors showed lower potency (3- to 10-folds) in suppressing T-cell function compared to B cells.

IFNα is mainly produced by pDCs upon activation of TLRs and up-regulated in peripheral blood of SLE patients. IFNα-induced genes have been shown to correlate with disease severity (18, 19) and PI3Kδ has been shown to mediate TLR9-induced IFNα release from pDCs (7). In agreement with this, we could show that MSC2360844 and MSC2364588 suppressed CpG oligodeoxynucleotide-induced TLR9-mediated IFNα production by pDCs (isolated from PBMC from healthy donors) in a concentration-dependent manner (Figure 2D). While both compounds showed lower potency in this assay (data not shown) compared to B- and T-cell inhibition, the relative potency of MSC2364588 over MSC2360844 is again preserved (five to sixfolds).

Taken together, both, MSC2360844 and MSC2364588, inhibited PI3Kδ-mediated functions in SLE relevant subsets of primary human cells. B cell function appeared most susceptible to PI3Kδ inhibition, followed by T-cells and pDCs. From the fact that the relative potency of our two compounds was preserved in all assays, cell types, and *in vitro* enzymatic activity (MSC2364588 is consistently 5–10 times more potent than MSC2360844), we conclude that the observed cellular effects are due to PI3Kδ-specific inhibition, not off-target activity.

### MSC2360844 and MSC2364588 dampened B- and T-cell functions in BioMap^®^ systems with a favorable safety profile

We next explored the effects of MSC2360844 and MSC2364588 in 12 BioMAP^®^ human primary cell co-culture systems (Section 3 in Supplemental Material). We used a broad concentration range (from 10 to 33.3 μM) to cover both on-target and possible off-target effects. No signs of cytotoxicity were observed at any concentration in any of the culture systems.

**Figure 3:**
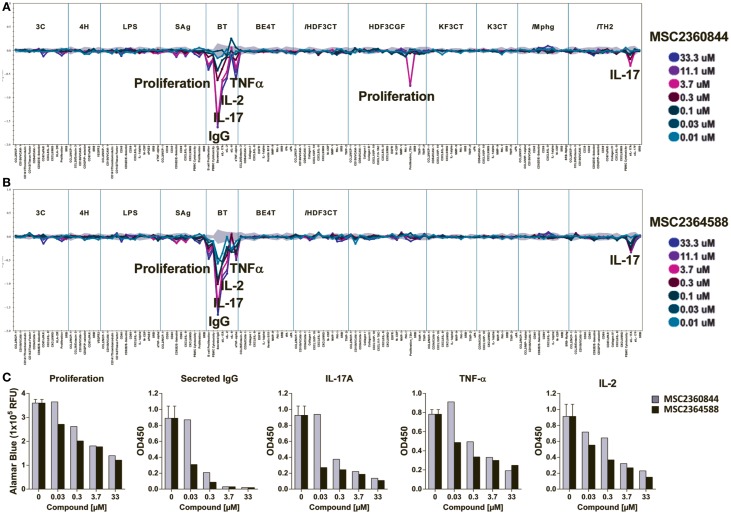
**MSC2360844 and MSC2364588 block B- and T-cell function without undesired side effects in 12 BioMAP^®^ systems**. MSC2360844 and MSC2364588 at indicated concentrations were tested in 12 human primary cell co-culture BioMAP^®^ systems with 137 readouts (Section 3 in Supplementary Material). Mixed primary cells were stimulated with environmental factors mimicking distinct disease or homeostatic conditions for 24 to 114 h, respectively. **(A,B)** Cellular activity profile of MSC2360844 in **(A)** and MSC2364588 in **(B)** in the 12 BioMAP^®^ systems. Levels of proteins were measured by ELISA and presented as log expression ratios [log10 (parameter value with test compound/parameter value of 0.1%DMSO)]. The gray area represents the 95% prediction interval of the 0.1% DMSO data. Significantly altered readouts are annotated. **(C)** Effects of MSC2360844 and MSC2364588 on annotated readouts from B–T assays. Level of proliferation was represented by relative fluorescent units (RFU) of Alamar Blue. Cytokines were measured by ELISA and presented as artificial unit of optical density at 450 nm (OD450).

Significant inhibitory effects of the two compounds were observed in the B–T system, a B cell/PBMC co-culture activated with anti-IgM and mild TCR stimulation for 84 h (Figures 3A,B). This system models both T-cell-dependent B cell activation and class-switching as would occur in a germinal center, and B-cell-dependent T-cell responses, such as cytokine release (13). In the B–T culture system, MSC2360844 and MSC2364588 significantly and dose-dependently inhibited B cell proliferation and IgG secretion, as well as IL-17A, IL-2, and TNFα release, but had no effect on IL-6 levels (Figures 3A,B). At concentrations ranging from 30 to 300 nM, MSC2364588 is more potent than MSC2360844 with respect to all inhibited readouts, showing estimated IC_50_ values close to those determined in isolated immune subsets (Figure 3C).

Confirming a direct role of PI3Kδ in T-cell activity, both compounds inhibited IL-17A release in the Th2 culture system, a co-culture of primary human umbilical vein endothelial cells (HUVEC) and Th2 blasts stimulated by TCR crosslinking and IL-2 (Figures 3A,B).

Unexpectedly, in a culture system modeling conditions of chronic inflammation and fibrosis (HDF3CGF), MSC2360844 at concentrations above 3.7 μM reduced the proliferation of human dermal fibroblasts stimulated with IL-1β, TNF-α, IFN-γ, EGF, bFGF, and PDGF-BB for 24 h. Since this inhibitory activity was neither dose-dependent nor observed with MSC2364588, it likely represents an off-target effect of MSC2360844 at very high concentrations. Intriguingly, this anti-proliferative effect was not observed in a similar culture system/HDF3CT, in which human dermal fibroblasts are stimulated with IL-1β, TNF-α, IFN-γ, and TGFβ for 24 h (Figures 3A,B).

Even at very high doses (33 μM), neither of our compounds exerted any significant effect in any of the other nine co-culture systems, which included cultures of HUVEC with or without PBMCs, M1 macrophages, human bronchial epithelial cells, or epidermal keratinocytes under conditions mimicking chronic inflammation (Figures 3A,B).

The activity profiles of MSC2360844 and MSC2364588 in these chronic inflammation relevant co-culture systems suggest that targeted PI3Kδ inhibition dampens specifically pro-inflammatory B- and T-cell functions without inducing cytotoxicity or undesired side effects in other cell types, which could limit their use in chronic inflammatory conditions.

### MSC2360844 and MSC2364588 inhibit pharmacodynamic biomarkers in human peripheral blood

The importance of monitoring relevant biomarkers to improve the efficiency of drug development is increasingly recognized. We identified and validated two PD biomarkers, pAkt and CD69, to assess target inhibition following *in vivo* administration of our PI3Kδ inhibitors. To this end, we quantified the activity of our compounds on BCR-mediated Akt phosphorylation (pAkt) and CD69 (early activation surface marker) up-regulation. Assays were done with whole blood from healthy donors.

**Figure 4:**
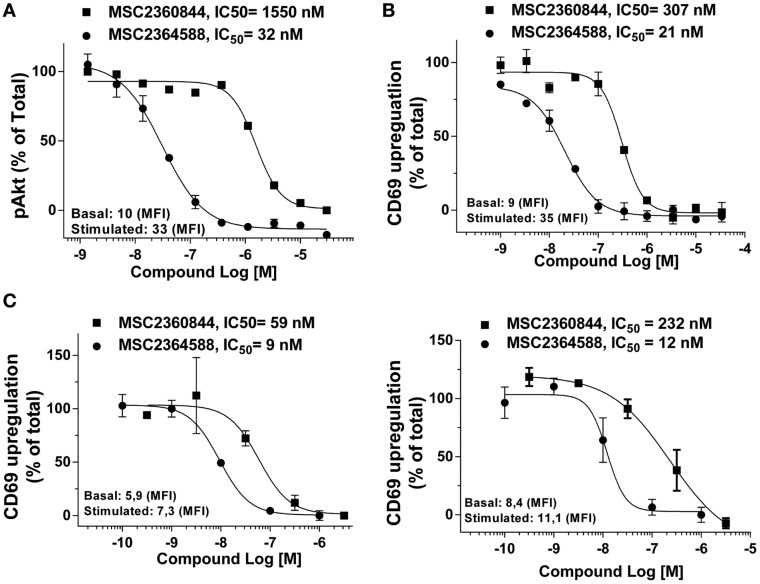
**MSC2360844 and MSC2364588 inhibit BCR-induced signaling and activation of CD19^+^ B cells in healthy donors and SLE patients**. **(A)** Peripheral blood from healthy donors were collected in heparinized tubes and stimulated with anti-IgM for 5 min. Intracellular pAkt gated on CD19^+^ cells were analyzed by flow cytometry. **(B)** Peripheral blood from healthy donors were collected in heparinized tubes and stimulated with anti-IgM for 18 h. CD69 up-regulation on CD19^+^ cells were analyzed by flow cytometry. Mean fluorescent intensity (MFI) from basal and stimulated conditions without inhibitor were used as 100% of activity. Data shown are mean ± SEM and are representative for at least five healthy donors. **(C)** PBMCs from two SLE patients were stimulated with anti-IgM for 18 h. CD69 up-regulation on CD19^+^ cells were analyzed by flow cytometry. Mean fluorescent intensity (MFI) from basal and stimulated conditions without inhibitor were used as 100% of activity.

MSC2364588 inhibited anti-IgM-induced pAkt in whole blood with IC_50_ of 32 nM (Figure 4A), similar to its potency in human Ramos cells (Figure 1C). MSC2360844, on the other hand, had IC_50_ of 1550 nM in whole blood (Figure 4A), approximately fivefold less potent compared to its IC_50_ in Ramos cells (Figure 1C). Besides the inherent difference in potency between the two compounds, these data also reflect their differential human plasma protein binding: while 100% of MSC2364588 is present in the unbound fraction in human plasma, only 20% of MSC2360844 is unbound (Table S1 in Supplementary Material). MSC2360844 and MSC2365488 also potently blocked anti-IgM-induced CD69 up-regulation on CD19^+^ B cells in whole blood, with IC_50_ of 307 and 21 nM, respectively, in line with their enzymatic and cellular potencies on PI3Kδ as well as plasma protein binding capacities (Figure 4B). Thus, MSC2360844 and MSC2365488 blocked PI3Kδ-mediated early B cell activation in peripheral blood from healthy donors in a concentration-dependent manner.

Anti-IgM induced a weak but consistent up-regulation of CD69 in PBMCs from SLE patients (Figure 4C) comparable to healthy donors (data not shown). PBMCs were recovered from cryopreservation and our unpublished data have shown that cryopreservation does not alter the responsiveness of PBMCs to anti-IgM stimulation. In a concentration-dependent manner, MSC2360844 and MSC2364588 abrogated CD69 up-regulation with IC_50_ of 59 and 9 nM, respectively, in one donor (Figure 4C, left), and 232 and 12 nM, respectively, in another (Figure 4C, right).

These data as well as the demonstrated technical feasibility of the assays qualify pAkt and CD69 as possible mechanistic PD biomarkers to determine the PK/PD relationship of PI3Kδ inhibitors in dose finding studies in clinical development.

### PK/PD correlation and modeling of MSC2360844 and MSC2364588 in mouse

**Figure 5:**
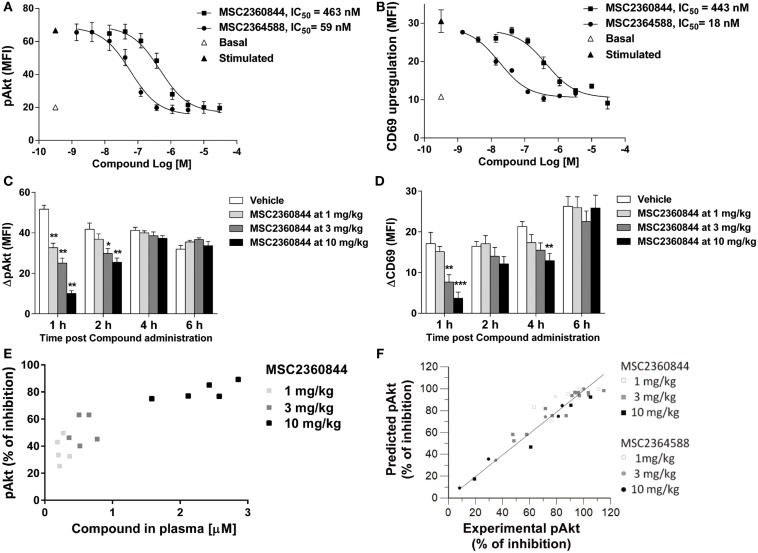
**MSC2360844 and MSC2364588 block the PD biomarkers in mouse and allow PK/PD modeling**. **(A,B)** In *in vitro* assays, blood from naïve C57Bl/6N mice was taken and stimulated *in vitro* with anti-IgD. In **(A)**, intracellular pAkt gated on B220^+^ cells was measured by flow cytometry and expressed as MFI. Data shown are mean ± SEM and are representative for three experiments. In **(B)**, surface CD69 gated on B220^+^ cells was measured by flow cytometry and expressed as MFI. Data shown are mean ± SEM and are representative for two experiments. **(C,D)** In *ex vivo* assays, MSC2360844 at indicated doses was administered by gavage to naïve C57Bl/6N mice at indicated time points before whole blood was taken and stimulated *in vitro* with anti-IgD. In **(C)**, intracellular pAkt was measured as in **(A)**. ΔpAkt was calculated by subtracting basal from stimulated signal. In **(D)**, surface CD69 was measured as in **(B)**. ΔCD69 was calculated by subtracting basal from stimulated signal. Data are shown as mean ± SEM with five mice per group. Data were analyzed by one-way ANOVA followed by Dunnett’s post-tests compared with vehicle group. **p < 0.01, ***p < 0.001. **(E)** MSC2360844 concentrations in plasma were plotted against percentage of pAkt inhibition at 1 h time point in the experiment described in **(C)**. **(F)** PK/PD model using percentage of pAkt inhibition readout based on data obtained from **(A,B)**. Points represent the average of pAkt (% of inhibition) of five animals at a single time or dose of a compound.

To establish the relationship between plasma exposure of MSC2360844 and MSC2364588 and their target coverage *in vivo*, we evaluated pAkt and CD69 PD biomarkers in mice. We first developed *in vitro* and *ex vivo* mouse whole blood assays for pAkt and CD69. Anti-IgD rather than anti-IgM was used to induce BCR activation in mice, because IgD activated a larger B cell population and the background noise was lower (data not shown). We then measured BCR activation-induced Akt phosphorylation in B220^+^ B cells (Figure 5A) and surface CD69 up-regulation on B cells *in vitro* (Figure 5B). MSC2360844 and MSC2364588 blocked both readouts completely in a concentration-dependent manner with average IC_50_s for MSC2360844: 361 nM for pAkt and 412 nM for CD69; and for MSC2364588: 35 nM for pAkt and 16 nM for CD69 (Figures 5A,B). In line with their enzymatic inhibitory activity, plasma protein binding (Table S1 in Supplementary Material) and human cellular activity (Figures 1 and 3), MSC2364588 was approximately 10-folds more potent than MSC2360844 in the mouse whole blood *in vitro* assay.

In agreement with the established *in vivo* PK profile in the mouse (Table S2 in Supplementary Material), the overall exposure level of MSC2360844 was approximately 10 times higher than that of MSC2364588 (Figures S2A,B in Supplementary Material). Oral administration of both compounds dose-dependently inhibited pAkt and CD69, with comparable ED_50_s of 3 mg/kg at the 1 h time point, and approximately 10 mg/kg at 2 h. At 4 h, the ED_50_s were further increased and at 6 h post administration almost no effect was detected (Figures 5C,D; Figures S2C,D in Supplementary Material). The higher exposure of MSC2360844 at equivalent dose compensated completely for its lower *in vitro* potency as compared to MSC2364588.

As shown in Figures S2A,B in Supplementary Material, a dose-dependent increase in exposure was observed, suggesting linear pharmacokinetics over the dose range studied. In addition, a direct relationship, i.e., absence of hysteresis, was observed when plasma concentrations were correlated to the PD effect, namely pAkt inhibition (Figure 5E; Figure S2E in Supplementary Material). These time-course and dose response data and the estimated PK/PD parameters (Table S2 in Supplementary Material) led to the establishment of an *I*_max_ mathematical model linking plasma concentrations to pAkt inhibition over time in which a correlation factor of 0.99 was calculated between predicted versus experimentally measured pAkt inhibition (Figure 5F). Thus, a quantitative relationship between dosing regimen, exposure, and PD effects of MSC2360844 and MSC2364588 was identified based on the correlation of *ex vivo* PD effects and *in vitro* potency, which allowed the extrapolation of a fitting mathematical PK/PD model.

### MSC2360844 ameliorates disease manifestations in a murine SLE model

**Figure 6:**
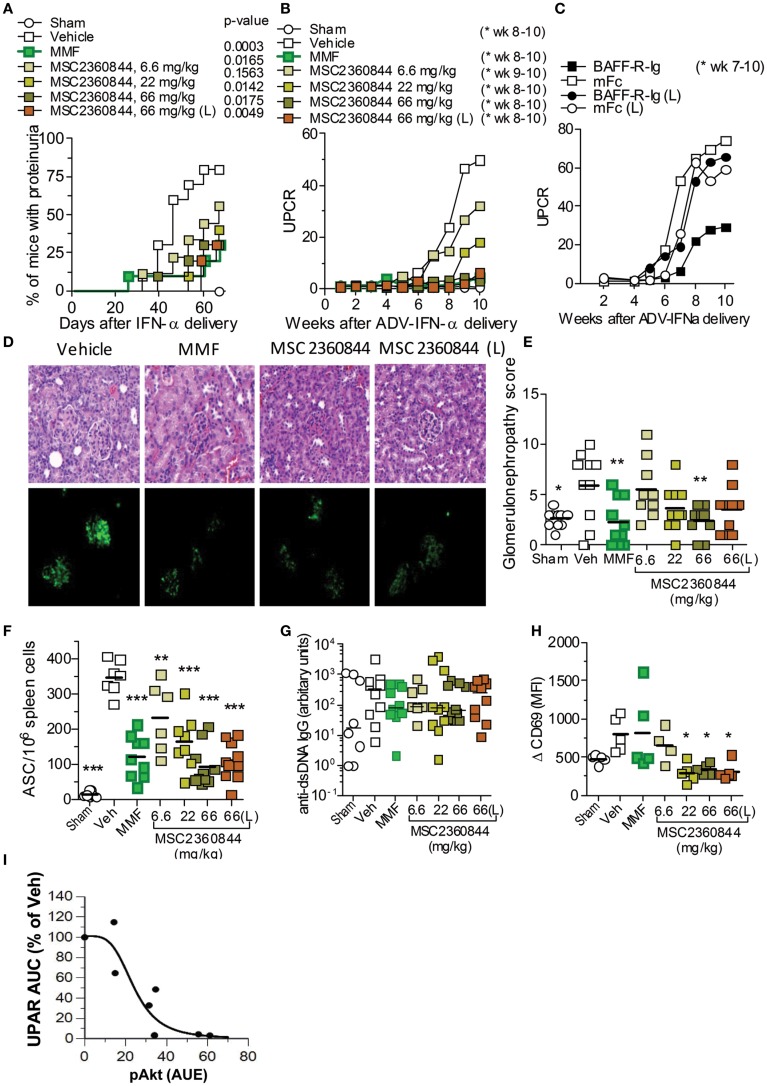
**MSC2360844 abrogates lupus nephritis development in accelerated NZB/W F1 SLE model**. **(A)** Incidence of proteinuria and **(B)** mean UPCR overtime in mice treated once daily with MSC2360844 at 6.6, 22, or 66 mg/kg, or MMF at 300 mg/kg with an early dosing regimen, or MSC2360844 at 66 mg/kg with a late dosing regimen (L). **(C)** UPCR over time in mice treated with BAFF-R-Ig or mFc as control. **(D)** Kidney sections obtained at the end of experiment were stained with H&E (upper panel) or immunofluorescence (lower panel) with anti-IgG2a FITC Ab. Magnification, 200×. **(E)** Histopathological scores in **(D)** (upper panel). Each dot represents one mouse. Bar represents mean value. **(F)** ASCs in the spleen measured with ELISpot at the end of experiment. Each dot represents one mouse. Bar represents mean value. **(G)** Levels of anti-dsDNA IgG at the end of experiment. Each dot represents one mouse. Bar represents mean value. **(H)** *Ex vivo* anti-IgD-induced CD69 up-regulation on B220^+^ cells in whole blood 1 h post last dosing after 2 week of MSC2360844 treatment. Each dot represents one mouse. Bar represents mean value. **(I)** Relationship between PD marker pAkt and UPCR. The pAkt PK/PD model was used to mimic pAkt response under efficacy experiment conditions. *p < 0.05, **p < 0.01, ***p < 0.001.

NZB/W F1 female mice are genetically predisposed for the development of a SLE-like phenotype including the production of autoantibodies (e.g., anti-dsDNA) and the development of glomerular nephritis (20). The onset of proteinuria in this model is at about week 40, but can be accelerated and synchronized by adenoviral delivery of IFN-α (ADV-IFNα) (21). Using a modification of the Mathian et al. protocol (21), we injected 1 × 10^8^ ADV-IFNα at 10 weeks of age. IFN-α peaked in the serum 7 days later and lasted for 1 week in NZB/W F1 female mice (data not shown). At week 2 post ADV-IFNα administration, these mice had detectable levels of anti-dsDNA Abs in the serum and developed proteinuria at week 4 (Figures 6A,B and data not shown) with no mortality by end of experiment at week 10, while sham mice (no IFNα treatment) were free of any sign of disease at this time (Figures 6A,B, and data not shown).

MSC2360844 exhibited higher oral availability in mouse (Table S2 in Supplementary Material), rat, dog, and monkey (data not shown) than MSC2364588. Human PK predication based on allometric scaling suggested that the half-life of MSC2360844 in human would be suitable for once daily dosing, whereas MSC2364588 was predicted to require twice daily dosing in human (data not shown). We therefore chose to test MSC2360844 in (i) early intervention (first autoantibodies in serum), starting at week 2 post ADV-IFNα delivery with once daily oral administration of 6.6, 22, or 66 mg/kg MSC2360844 or MMF at 300 mg/kg (equivalent to clinically efficacious dose) as positive control, and in (ii) late intervention (onset of proteinuria), starting at week 4 post ADV-IFNα delivery (L) with once daily oral administration of 66 mg/kg MSC2360844. In early intervention, all doses of MSC2360844 significantly reduced proteinuria incidence and severity in a dose-dependent manner compared to vehicle and as determined by UPCR (Figures 6A,B). Mice treated with 66 mg/kg MSC2360844 were almost completely protected against proteinuria, equivalent to MMF-treated mice. Even in late intervention, 66 mg/kg MSC2360844 almost completely abrogated proteinuria (Figures 6A,B). However, inhibition of BAFF (using BAFFR-Ig) was effective in this model only following early but not late intervention as determined by UPCR (Figure 6C).

The protective effects of MSC2360844 were confirmed by histological analysis of the kidneys at the end of the experiment, showing dose-dependent reduction of inflammation, glomerular damage, and sclerosis (Figures 6D,E). Consistently, levels of pro-inflammatory cytokines, IL-6, TNFα, GM-CSF, and IL-1β, and IgG (immune-complex) deposition in the kidney were also decreased in a dose-dependent manner (Figure S3 in Supplementary Material; Figure 6D).

Compared to sham mice, at week 10, ADV-IFNα-treated NZB/W F1 mice showed dysregulation of immune cells in the spleen, with a significant increase in the number of plasma cells, GL-7^+^IgG^−^ GC B cells, IgG^+^ class-switched B cells, and activated CD4^+^ and CD8^+^ T-cells, while naïve CD4^+^ and CD8^+^ T-cell numbers were reduced (Table 2). MSC2360844 treatment, dose-dependently, restored homeostatic levels by reducing the numbers of activated GL-7^+^IgG^−^GC B cells, IgG^+^ class-switched B cells, and plasma cells, as well as activated CD4^+^ and CD8^+^ T-cells, and by increasing naïve CD4^+^ and CD8^+^ T-cells to the level of sham mice (Table 2). Consistent with reduced plasma cell numbers, antibody-secreting cells (ASCs) in the spleen were also decreased significantly in a dose-dependent manner by MSC2360844 treatment. Both, early and late intervention, 66 mg/kg MSC2360844 groups showed equivalent effects to MMF (Figure 6F).

**Table 2:** **Numbers of lymphocyte subsets in spleen at end of the treatment**.

No. of cells (×10^6^) (mean ± SD)	Sham	Vehicle	MMF	MSC2360844 6.6 mg/kg	MSC2360844 22 mg/kg	MSC2360844 66 mg/kg	MSC2360844 66 mg/kg (L)
Total splenocytes	33.34* (±12.53)	51.94 (±19.70)	46.50 (±25.17)	51.90 (±25.17)	46.64 (±21.37)	38.41 (±11.39)	37.79 (±15.17)
B220^+^CD19^+^ (total B cells)	13.28* (±7.16)	24.92 (±9.89)	20.65 (±6.54)	24.52 (±13.69)	21.75 (±11.10)	18.07 (±5.91)	16.08 (±8.59)
CD138^+^ (plasma cells)	0.22* (±0.23)	0.84 (±0.32)	0.43 (±0.28)	0.67 (±0.50)	0.52 (±0.54)	0.23* (±0.13)	0.27* (±0.28)
GL-7^+^IgG^−^ (GC B cells)	0.18 (±0.20)	0.46 (±0.22)	0.23 (±0.16)	0.49 (±0.18)	0.42 (±0.24)	0.18 (±0.11)	0.20 (±0.15)
IgG^+^ (class-switched B cells)	0.13 (±0.07)	0.24 (±0.18)	0.17 (±0.05)	0.18 (±0.13)	0.19 (±0.08)	0.12* (±0.03)	0.13 (±0.07)
CD3^+^ (total T-cells)	16.26 (±3.83)	15.90 (±4.75)	21.05 (±6.83)	19.44 (±5.39)	21.22 (±5.95)	17.90 (±5.57)	16.29 (±4.69)
CD4^+^ CD44ow^low^ CD62Ligh^high^ (naive CD4^+^ T-cells)	5.39 (±1.32)	3.50 (±1.53)	6.77* (±2.57)	6.03 (±1.64)	7.08* (±1.35)	6.59* (±2.43)	5.61 (±1.62)
CD4^+^ CD44igh^high^ CD62Low^low^ (activated CD4^+^ T-cells)	3.38* (±1.82)	7.23 (±3.00)	5.24 (±2.05)	6.10 (±4.51)	5.30 (±3.19)	3.03* (±1.31)	3.44 (±2.23)
CD8^+^ CD44ow^low^ CD62Ligh^high^ (naive CD8^+^ cells)	4.13* (±1.12)	2.07 (±0.80)	5.06* (±1.76)	3.78 (±0.95)	4.89* (±1.89)	5.15 (±1.74)	4.19* (±1.37)
CD8^+^ CD44igh^high^ CD62Low^low^ (activated CD8^+^ T-cells)	0.18* (±0.08)	0.46 (±0.25)	0.23 (±0.09)	0.33 (±0.21)	0.34 (±0.28)	0.21 (±0.09)	0.26 (±0.22)

Statistical analysis; one-way ANOVA followed by Dunnett’s test versus vehicle group. *p < 0.05.

There was a tendency of serum anti-dsDNA IgG reduction following early treatment with both, MSC2360844 or MMF, although this was neither statistically significant nor dose-dependent and no effect was observed following late treatment with MSC2360844 (Figure 6G). These data indicate dissociation between the level of anti-dsDNA Ab in the serum, ASCs in the spleen and immune-complex formation and deposition (Figure 6D).

Overall, MSC2360844 is equivalent to 300 mg/kg MMF at a dose between 22 and 66 mg/kg with respect to modulation immune cell responses, protection of kidney function, and reduction of inflammation and tissue damage (Table 2; Figure 6A,B,D,G; Figure S3 in Supplementary Material).

To exclude the possibility that the beneficial effects demonstrated with MSC2360844 were due to off-target activity at peak serum concentration following once daily doses, we also tested MSC2360844 (data not shown) and MSC2364588 (Figure S4 in Supplementary Material) at half daily dose given twice daily in early intervention. Equivalent results were obtained, suggesting that MSC2360844 and MSC2364588 block proteinuria progression through selective PI3Kδ inhibition. Encouragingly, no drug-related body weight loss was observed throughout these studies (data not shown).

To further describe the PK/PD relationship of MSC2360844 under disease conditions, we tested the PD response in the SLE model. Mice were treated once daily for 2 weeks with MSC2360844 in early intervention. One hour after the last dosing, blood was taken and stimulated *ex vivo* with anti-IgD. B220^+^ B cells were then assessed for CD69 up-regulation. B cells form MSC2360844-treated mice showing a significant, dose-dependent reduction of CD69 surface expression, whereas B cells from MMF-treated mice were indistinguishable from untreated mice (Figure 6H), indicating that MSC2360844 inhibited BCR-mediated early activation of B cells *in vivo* whereas MMF affects B cell function via a different pathway.

The established PK/PD model was next applied to simulate pAkt response following repeated inhibitor treatment in the SLE model. Areas under the effects of response (AUE) on pAkt over the whole treatment period were estimated and correlated to UPCR. A steep correlation between PD and efficacy was observed with hill number of 3.9 with AUE50 at 24 ± 5% inhibition of pAkt over 24 h. These data suggest dosing of PI3Kδ inhibitor with 24% of target coverage over 24 h is sufficient to achieve 50% efficacy, and a complete inhibition of the disease can be achieved by 50% target coverage over time in this SLE model (Figure 6I).

In summary, intervention with PI3Kδ inhibitors after break down of tolerance, either at the onset of serum anti-dsDNA Ab or the onset of proteinuria, significantly blocked disease progression and kidney damage, similar to the benchmark immunosuppressant drug MMF, in the ADV-IFNα-accelerated NZB/W F1 SLE model.

## Discussion

We compared head-to-head two novel PI3Kδ inhibitors, MSC2360844 and MSC2364588, in single cell type cultures and BioMAP^®^ co-culture systems. We confirmed a central role of PI3Kδ in human B- and T-cell functions in conditions mimicking autoimmune disease pathology without detectable signs of undesired side effects. We also demonstrated that selective inhibition of PI3Kδ blocked disease progression and kidney damage in a mouse model of SLE/lupus nephritis. These protective effects correlated with reduced B- and T-cell activity, compound target coverage, and levels of PD markers, pAkt, and CD69.

Our PI3Kδ inhibitors are derived from a thiochroman series. MSC2360844 exhibited better DMPK properties but relatively lower selectivity versus other PI3K isoforms (namely, PI3Kβ), whereas MSC2364588 showed higher potency and selectivity but relatively poorer DMPK properties. Evaluation of the two compounds side-by-side in all assays allowed us to exemplify PI3Kδ-specific functional effects.

The roughly 10-fold difference between MSC2360844 and MSC2364588 in PI3Kδ inhibition, as shown in the enzymatic assay, was consistently reproduced in all functional cell-based assays (containing no or low plasma protein). This suggests strongly that the cellular activity of both compounds was on-target. Taking into account differential plasma protein binding, clearance, and oral bioavailability of the two compounds, this relationship was also preserved in whole blood and *in vivo*. These data confirm MSC2360844 and MSC2364588 as selective and potent PI3Kδ inhibitors *in vitro* and *in vivo*.

In line with the reported roles of PI3Kδ, our compounds exhibited strong effects in human B- and T-cell and pDC functional assays. In addition, they were also efficacious in suppressing Ab and pro-inflammatory cytokine production in the BioMAP^®^ B–T co-culture system, suggesting PI3Kδ inhibition can block interdependent B- and T-cell responses under pathophysiological conditions in immune-mediated inflammatory diseases.

IFNα-inducible gene signature has been associated with patients with severe SLE (18, 19) and exogenous IFNα can induce autoimmunity in humans, including SLE (22). We chose to evaluate the *in vivo* efficacy of our compounds in an IFNα-accelerated murine SLE model. Exposure of lupus-prone NZB/W F1 mice to IFNα leads to an accelerated and synchronized disease onset, increased disease severity, and lower survival rate (21). While untreated NZB/W F1 mice go into long-term remission following a short course of combined cyclophosphamide, CTLA4-Ig and anti-CD40L treatment, adenovirus (ADV)-IFNα-accelerated NZB/W F1 mice are only transiently affected (23), demonstrating the severity of the disease in this model, possibly representing the subset of SLE patients with IFNα-gene signature. We adapted the model form Mathian et al. with a modified protocol applying a single i.v. injection of 1 × 10^8^ IU/100 μl of ADV-IFNα which resulted in the appearance of serum anti-dsDNA antibodies at week 2, proteinuria at week 4, and no mortality by week 10. Interestingly, at the time of anti-dsDNA appearance, the expansion of ASCs and a small number of IL-17- but not IFNγ- or TNFα-producing T-cells were detectable; whereas at the onset of proteinuria, both ASCs and a large number of IL-17-, IFNγ-, and TNFα-producing T-cells were present in the spleen (Figure S5 in Supplementary Material). Likewise, patients with SLE have increased numbers of IL-17-producing cells and an elevated level of IL-17 in the serum, and IL-17-producing cells in affected kidneys (17, 24). It is possible that IL-17-producing cells, as opposed to other T-cell subsets, are early participants in mounting the immune response in peripheral lymphoid tissues (25, 26) and target organs in these patients. Recently, it has been shown that increased Th17 cells correlates with IFN type 1-inducible signature in SLE patients (27). Taken together, the ADV-IFNα-accelerated NZB/W F1 mouse model likely represents a subset of SLE patients with severe disease activity, IFNα-signature, and engaged IL-17 pathway in addition to active B cell involvement with elevated anti-dsDNA Ab titers.

Although both B- and T-cells are thought to be key players, our understanding of the molecular pathophysiology of SLE and lupus nephritis is still incomplete. Belimumab (anti-BAFF/BLYSS Ab), the first new approved drug for SLE in more than 50 years, appears to show modest efficacy and is indicated as add-on therapy in patients with active SLE receiving standard therapy, thus excluding active lupus nephritis and neuropsychiatric lupus (3). MMF, although generally effective, both as induction and maintenance therapy, for patients with severe SLE manifestations including lupus nephritis, comes with long-term safety concerns (25). Comparable to the efficacy of MMF and a BAFF blocker, our compounds dose-dependently prevented the development of proteinuria and kidney lesions in the ADV-IFNα-accelerated lupus model when administered at the onset of autoantibody production. Interestingly, at the onset of proteinuria, only PI3Kδ inhibitors and MMF but not BAFF blocker was efficacious in preventing disease progression. Our data therefore suggest that, despite the presence of IL-17-producing T-cells, B cells play a central role in the early phase of the disease, which can be blocked with B cell modulators; at the time when kidney function becomes compromised, the disease is driven by both B- and T-cells and thus only therapeutics that block both cell activities, including cytokine production, such as PI3Kδ inhibitors or MMF, can be effective.

Anti-dsDNA Abs are routinely measured to monitor disease activity in SLE and have been associated with severe manifestations of lupus such as glomerulonephritis (28). MSC2360844 treatment in the IFNα-accelerated SLE model led to a decrease in numbers of ASCs (Figure 6F), CD138^+^ plasma cells, IgG^+^ class-switched B cells (Table 2) in the spleen as well as a decrease in IgG2 depositions in the kidney (Figure 6D). However, no statistically significant reduction of anti-dsDNA Ab serum levels was observed as is the case of MMF treatment (Figure 6G). The discrepancy between tissue and blood Ab levels may be attributed to: (1) the fact that IFNα-accelerated NZBxW F1 mice are more resistant to treatment than other SLE models (23), probably because IFNα is potentiating T and B cell functions including class-switch and IgG production (29, 30); (2) the possibility that different B cell compartments are the source of IgG deposited in the kidney and serum anti-dsDNA Abs; (3) the possibility that the IgG deposition in the kidney is less dependent on serum antibody levels, than it is on other unknown factors; (4) a too short observation period under treatment given a long circulating Ab half-life.

Similarly, elevated serum anti-dsDNA Ab levels have been detected in patients in the absence of any SLE flare or detectable disease activity (31). Likewise, the clinical efficacy of rituximab in SLE correlates with B cell depletion and precedes by several months any decline in autoantibodies (32). Our data further support that serum anti-dsDNA Ab titers do not always correlate with immune-complex deposition in the kidney or lupus nephritis disease activity following short-term immune modulation.

It is noteworthy that in this disease model, PI3Kδ inhibition restored homeostasis of lymphocyte populations in the spleen: hyperactive B-, T-, and plasma cells were reduced, whereas naïve B- and T-cell numbers were restored to normal levels. This is in contrast to mice deficient for PI3Kδ function showing reduced numbers of B cell precursors and mature B cells in the spleen (16, 33). These data suggest that PI3Kδ inhibition ameliorates SLE-like disease via immunomodulation rather than immunosuppression.

It should be mentioned that dysfunction of Tregs and AICD of T-cells have also been observed in SLE patients (8, 34, 35). Since PI3Kδ has been demonstrated to be important for Treg development and function in mice (36) and confers resistance to T-cell AICD in SLE patients (8), future studies addressing the effects of these PI3Kδ inhibitors on AICD and especially on human Tregs are warranted.

PK/PD relationship studies during pre-clinical drug development have the potential to significantly accelerate and reduce the cost of clinical development by providing early dose guidance for safety and efficacy (37). The consistent application of pAkt and CD69 as PI3Kδ PD biomarkers in mouse and human, a whole blood assay format throughout our pre-clinical studies not only validated their biological relevance, but also allowed us to determine the exposure–effect relationship and the correlation between PI3Kδ inhibition and efficacy in the disease model. Our data showed that an overall target coverage of 24% was sufficient to achieve 50% of clinical efficacy, while 50% target coverage was sufficient to achieve full clinical efficacy potential in the ADV-IFNα-accelerated lupus model. This is further supported by a study in which PI3Kδ activity was genetically attenuated to 50% in Lyn-deficient mice. These mice, which normally develop spontaneously a severe form of lupus nephritis, now showed significantly reduced kidney pathology (9). Thus, our data provide measurable PD markers and guidance for clinical study design aiming at achieving anticipated efficacy while keeping potential safety issues due to excessive dosing at bay.

While preparing this manuscript, two recent publications report that a novel PI3Kδ and PI3Kγ dual inhibitor suppresses anti-dsDNA Ab development and kidney dysfunction in the NZB/W F1 lupus nephritis model (38). This protective effect may be due to PI3Kδ inhibition since a selective PI3Kδ inhibitor IC87114 decreases autoantibody production and kidney pathology in another SLE-like model, BXSB mice (39). Our data largely corroborate these findings, except that we did not observe a significant dose-dependent reduction of anti-dsDNA Ab in serum after 10 weeks of treatment. The discrepancy may due to different SLE models and/or different treatment regimens. It is possible that prolonged MSC2360844 treatment would lead to a significant decrease of serum anti-dsDNA titer (32). Taken together, PI3Kδ inhibition demonstrated efficacy in three SLE mouse models; these data collectively support a critical role of PI3Kδ in the pathogenesis of SLE/lupus nephritis.

In summary, targeted PI3Kδ inhibition dampens kidney disease manifestation in SLE models through down-regulation of abnormal B-, T-, and pDC cell function and re-establishes immune cell homeostasis. We propose PI3Kδ inhibitors as potential therapeutics for treating patients with SLE and lupus nephritis, and possibly other autoimmune diseases.

## Author Contributions

Philipp Haselmayer, Lisa Bruns, and Roland Grenningloh generated NZB/W F1 experiment data. Montserrat Camps, Caroline Waltzinger, Carole Jond-Necand, Denise Gretener, and Maria Stella Lombardi generated human cellular experiment data. Mathilde Muzerelle generated compounds and enzymatic activity data. Samer El Bawab and Nada Abla generated PK and PK/PD modeling data. Mark A. Polokoff generated BioMap^®^ data. Hong Ji, Marilène Gaudet, Audrey Benoit, Dominique Bertschy Meier, and Catherine Martin generated mouse PD data. All authors participated in research design and interpretation of the data. Pascale Gaillard, Jørgen Søberg Petersen, and Christoph Ladel supervised the project. Hong Ji, Philipp Haselmayer, Montserrat Camps, and Mathilde Muzerelle drafted the paper. Hong Ji supervised the manuscript preparation. All authors provided critical input for the revision and gave consent for the final version, and agreed to be accountable for all aspects of the work.

## Conflict of Interest Statement

All authors, with the exception of Mark A. Polokoff, were employed by Merck Serono at the time of these studies. Montserrat Camps, Mathilde Muzerelle, Caroline Waltzinger, Mark A. Polokoff, Carole Jond-Necand, Marilène Gaudet, Audrey Benoit, Dominique Bertschy Meier, Catherine Martin, Denise Gretener, Maria Stella Lombardi, Jørgen Søberg Petersen, and Hong Ji declare no further conflicting financial interests. Philipp Haselmayer, Samer El Bawab, Lisa Bruns, Roland Grenningloh, and Christoph Ladel are currently employed by Merck Serono.

## Supplementary Material

The Supplementary Material for this article can be found online at http://www.frontiersin.org/Journal/10.3389/fimmu.2014.00233/abstract
