# Supplementary material for: Characterization of Novel PI3Kδ Inhibitors as Potential Therapeutics for SLE and Lupus Nephritis in Pre-Clinical Studies
Source: Front Immunol. 2014 May 22;5:233. doi: 10.3389/fimmu.2014.00233 (PMC4033217; doi:10.3389/fimmu.2014.00233)
Supplement: Supplementary file 1 [file Presentation1.PDF]

Supplementary Material

Characterization of novel PI3Kδ inhibitors as potential therapeutics for SLE and lupus nephritis in pre-clinical studies

Philipp Haselmayer[1,10,%], Montserrat Camps[2,11,%], Mathilde Muzerelle[3,%,#], Samer El Bawab[4], Caroline Waltzinger[2,11,†], Lisa Bruns[1,10], Nada Abla[5], Mark A. Polokoff[6], Carole Jond-Necand[2,11], Marilène Gaudet[7,11], Audrey Benoit[7,11], Dominique Bertschy Meier[7,11], Catherine Martin[7,11], Denise Gretener[8], Maria Stella Lombardi[2,11,‡], Roland Grenningloh[9], Christoph Ladel[10], Jørgen Søberg Petersen[11§], Pascale Gaillard[3¤], Hong Ji[7,11,*]

[1]Department of In Vivo Pharmacology, Merck Serono SA, Darmstadt, Germany
[2]Department of Cellular Immunology, Merck Serono SA, Geneva, Switzerland
[3]Department of Chemistry, Merck Serono SA, Geneva, Switzerland
[4]DMPK, Non-Clinical Department, Merck Serono SA, Darmstadt, Germany
[5]DMPK, Non-Clinical Department, Merck Serono SA, Geneva, Switzerland
[6]DiscoveRx Corporation, BioSeek® division, South San Francisco, CA, USA
[7]Department of Early PK/PD Biomarker, Merck Serono SA, Geneva, Switzerland
[8]Department of Screening, Merck Serono SA, Geneva, Switzerland
[9]Department of In Vivo Pharmacology, EMD Serono, Billerica, MA, USA
[9]Biologics and Immunology Platform, Merck Serono SA, Darmstadt, Germany
[10]Biologics and Immunology Platform, Merck Serono SA, Geneva, Switzerland

*** Correspondence:** Hong Ji. Current address: Department of Translational Immunology, Novo Nordisk A/S, Novo Nordisk Park, Måløv, 2760, Denmark
hji@novonordisk.com

%: P.H., M.C. and M.M. contributed equally to this paper.
#: MM´s current address: Debiopharm Research and Manufacturing S.A., Martigny, Switzerland
†: CW´s current address: Roche Glycart AG, Schlieren, Switzerland
‡: MSL´s current address: University of Geneva, Geneva, Switzerland
§: JSP´s current address: Novo Nordisk A/S, Måløv, Denmark
¤: PG´s current address: ALAXIA SAS, Lyon, France

1. Supplementary Tables

1.1. Supplementary Table 1. *In vitro* DMPK properties

	In vitro Clint (h/m) (µL/min/mg prot.)	Caco-2 Papp (x10^{-6} cm/s) /efflux ratio	uPPB % (h/m)
MSC2360844	< 10 / 18	11 / 1.2	20 / 33
MSC2364588	21 / 35	2.4 / 1.6	100 /65

CLint: intrinsic clearance in liver microsomes. h/m: human/mouse. Papp: apparent permeability. uPPB: unbound plasma protein binding.

1.2. Supplementary Table 2. Estimated *in vivo* PK and PD parameters

	MSC2360844	MSC2364588
PK Parameters		
CL/F (L/h/kg)	2.7	25.1
V/F (L/kg)	2.8	5.8
Ka (h-1)	9.3	1.6
$T_{1/2}$ (h)	1.7	0.6
PD Parameters		
IC_{50} (nM)	770	47
Hill	1.37	1.37

CL: Clearance; F: Absolute bioavailability; V: Central volume of distribution; Ka: First-order absorption rate constant

2. Supplementary Figures

2.1. Supplementary Figure 1

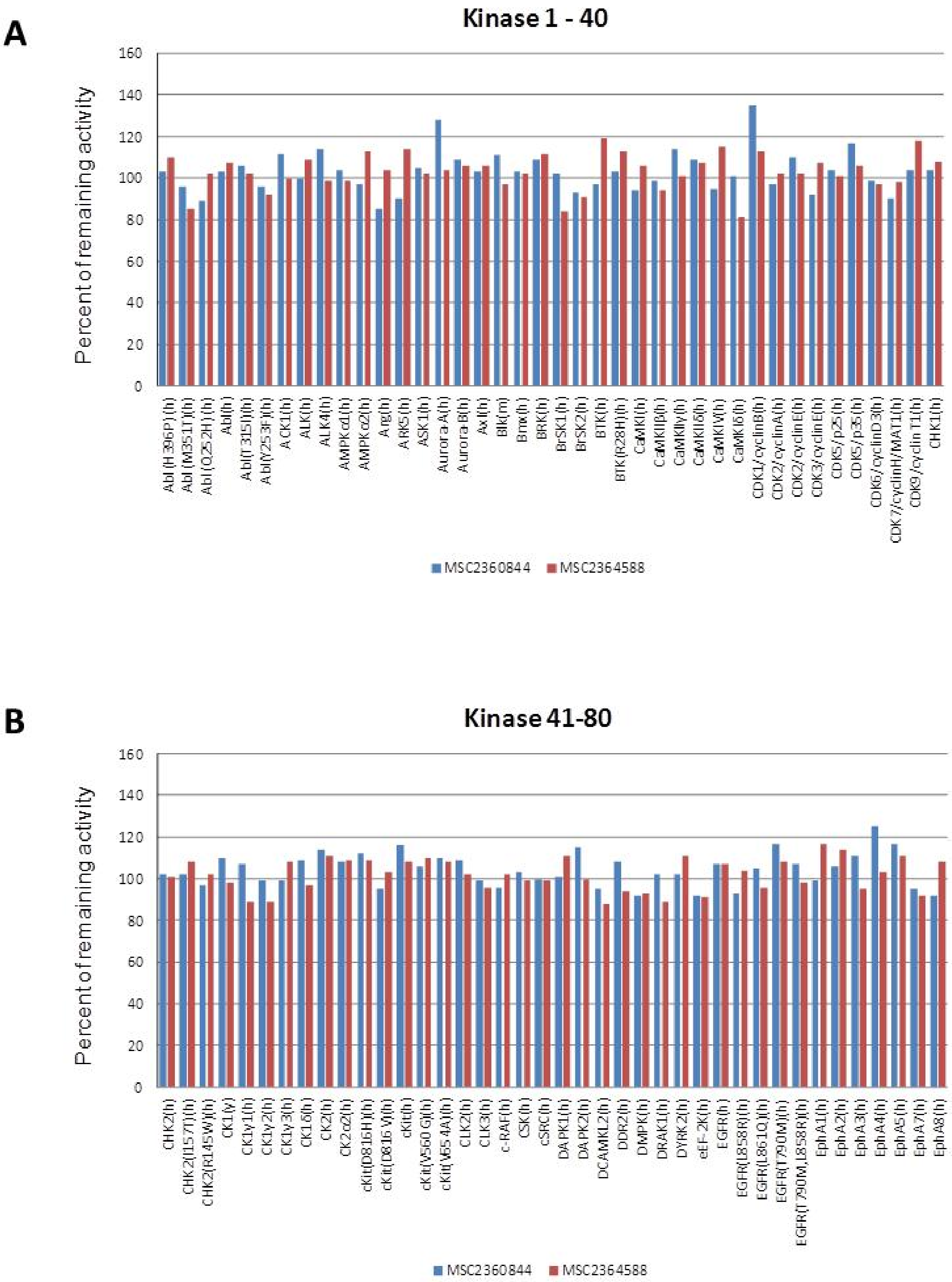
A
Kinase 1 - 40
Percent of remaining activity
160
140
120
100
80
60
40
20
0
Abl (H396P) (h)
Abl (M351T)(h)
Abl (Q252H) (h)
Abl(h)
Abl(T315I)(h)
Abl(Y253F)(h)
ACK1(h)
ALK(h)
ALK4(h)
AMPKα1(h)
AMPKα2(h)
Arg(h)
ARK5(h)
ASK1(h)
Aurora-A(h)
Aurora-B(h)
Axl(h)
Blk(m)
Bmx(h)
BRK(h)
BrSK1(h)
BrSK2(h)
BTK(h)
BTK(R28H)(h)
CaMKI(h)
CaMKIIβ(h)
CaMKIIγ(h)
CaMKIIδ(h)
CaMKIV(h)
CaMKIδ(h)
CDK1/cyclinB(h)
CDK2/cyclinA(h)
CDK2/cyclinE(h)
CDK3/cyclinE(h)
CDK5/p25(h)
CDK5/p35(h)
CDK6/cyclinD3(h)
CDK7/cyclinH/MAT1(h)
CDK9/cyclin T1(h)
CHK1(h)
MSC2360844
MSC2364588
B
Kinase 41-80
Percent of remaining activity
160
140
120
100
80
60
40
20
0
CHK2(h)
CHK2(I157T)(h)
CHK2(R145W)(h)
CK1(y)
CK1γ1(h)
CK1γ2(h)
CK1γ3(h)
CK1δ(h)
CK2(h)
CK2α2(h)
cKit(D816H)(h)
cKit(D816V)(h)
cKit(h)
cKit(V560G)(h)
cKit(V654A)(h)
CLK2(h)
CLK3(h)
c-RAF(h)
CSK(h)
cSRC(h)
DAPK1(h)
DAPK2(h)
DCAMKL2(h)
DDR2(h)
DMPK(h)
DRAK1(h)
DYRK2(h)
eEF-2K(h)
EGFR(h)
EGFR(L858R)(h)
EGFR(L861Q)(h)
EGFR(T790M)(h)
EGFR(T790M,L858R)(h)
EphA1(h)
EphA2(h)
EphA3(h)
EphA4(h)
EphA5(h)
EphA7(h)
EphA8(h)
MSC2360844
MSC2364588

C

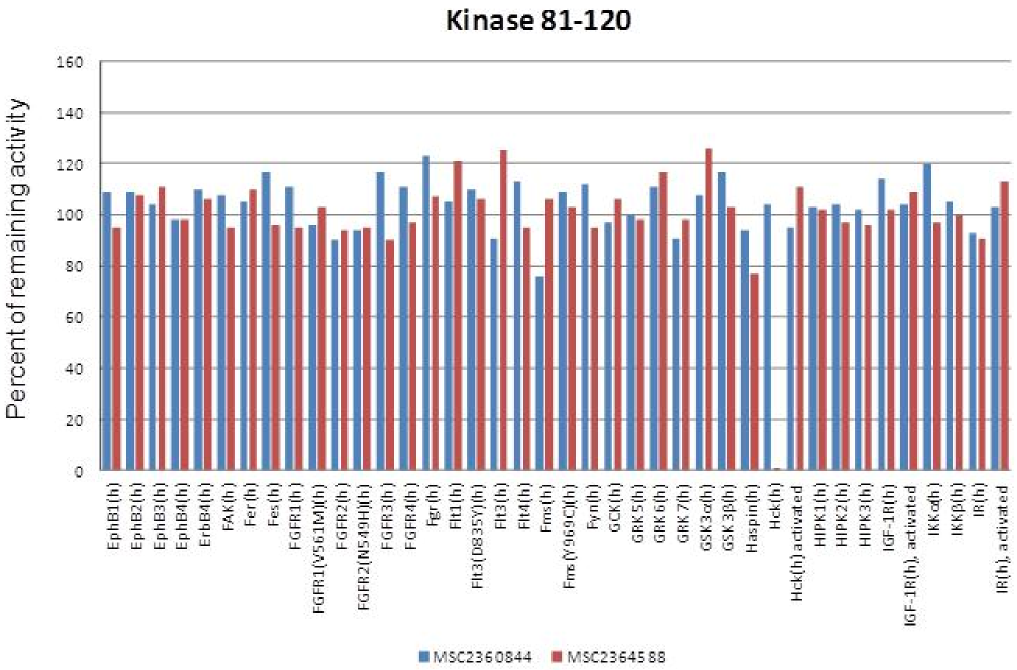
Kinase 81-120
Percent of remaining activity
160
140
120
100
80
60
40
20
0
EphB1(h)
EphB2(h)
EphB3(h)
EphB4(h)
ErbB4(h)
FAK(h)
Fer(h)
Fes(h)
FGFR1(h)
FGFR1(V561M)(h)
FGFR2(h)
FGFR2(N549H)(h)
FGFR3(h)
FGFR4(h)
Fgr(h)
Flt1(h)
Flt3(D835Y)(h)
Flt3(h)
Flt4(h)
Fms(h)
Fms(Y969C)(h)
Fyn(h)
GCK(h)
GRK5(h)
GRK6(h)
GRK7(h)
GSK3α(h)
GSK3β(h)
Haspin(h)
Hck(h)
Hck(h) activated
HIPK1(h)
HIPK2(h)
HIPK3(h)
IGF-1R(h)
IGF-1R(h), activated
IKKα(h)
IKKβ(h)
IR(h)
IR(h), activated
MSC2360844
MSC2364588

D

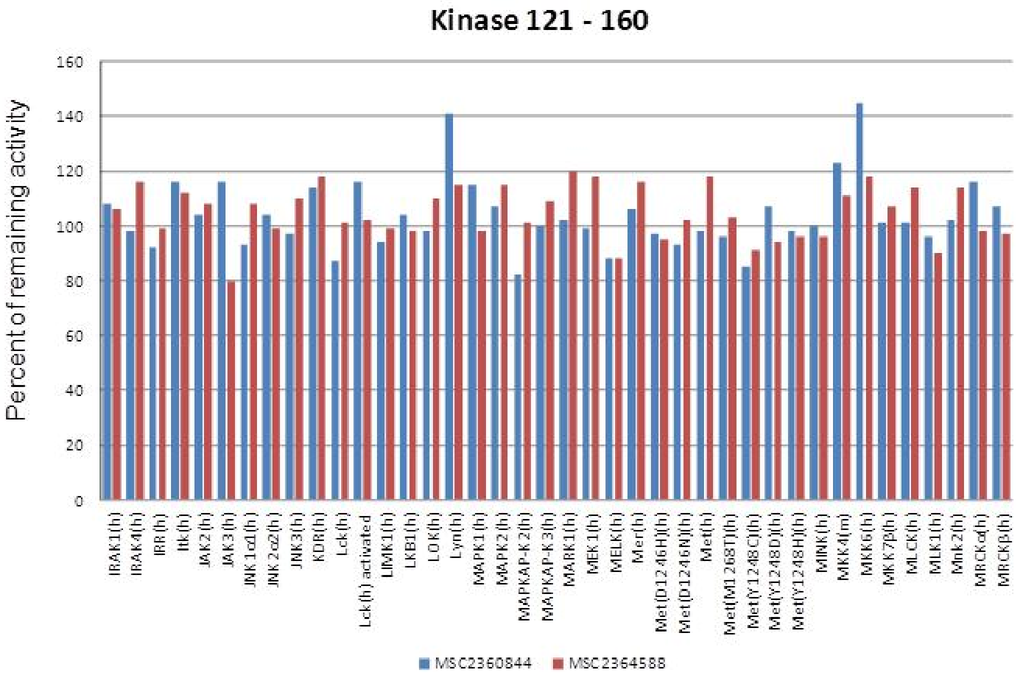
Kinase 121 - 160
Percent of remaining activity
160
140
120
100
80
60
40
20
0
IRAK1(h)
IRAK4(h)
IRR(h)
Itk(h)
JAK2(h)
JAK3(h)
JNK1α1(h)
JNK2α2(h)
JNK3(h)
KDR(h)
Lck(h)
Lck(h) activated
LIMK1(h)
LKB1(h)
LOK(h)
Lyn(h)
MAPK1(h)
MAPK2(h)
MAPKAP-K2(h)
MAPKAP-K3(h)
MARK1(h)
MEK1(h)
MELK(h)
Mer(h)
Met(D1246H)(h)
Met(D1246N)(h)
Met(h)
Met(M1268T)(h)
Met(Y1248C)(h)
Met(Y1248D)(h)
Met(Y1248H)(h)
MINK(h)
MKK4(m)
MKK6(h)
MKK7β(h)
MLCK(h)
MLK1(h)
Mnk2(h)
MRCKα(h)
MRCKβ(h)
MSC2360844
MSC2364588

E

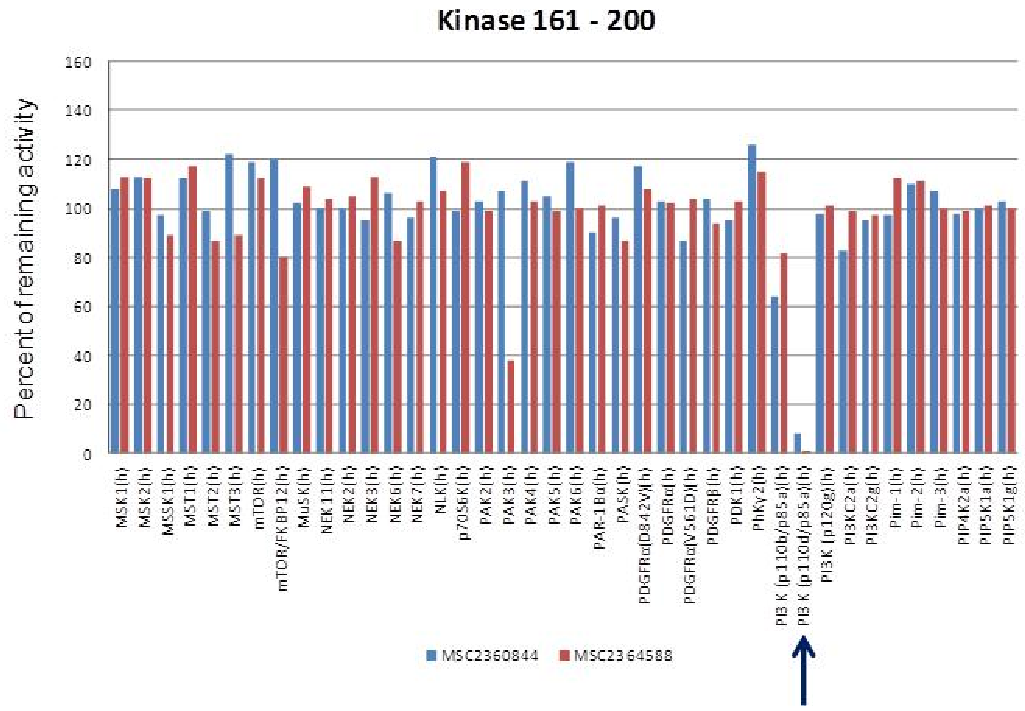
Kinase 161 - 200
Percent of remaining activity
0
20
40
60
80
100
120
140
160
MSK1(h)
MSK2(h)
MSSK1(h)
MST1(h)
MST2(h)
MST3(h)
mTOR(h)
mTOR/FKBP12(h)
MuSK(h)
NEK11(h)
NEK2(h)
NEK3(h)
NEK6(h)
NEK7(h)
NLK(h)
p70S6K(h)
PAK2(h)
PAK3(h)
PAK4(h)
PAK5(h)
PAK6(h)
PAR-1Bα(h)
PASK(h)
PDGFRα(D842V)(h)
PDGFRα(h)
PDGFRα(V561D)(h)
PDGFRβ(h)
PDK1(h)
PhKγ2(h)
PI3K (p110b/p85a)(h)
PI3K (p110d/p85a)(h)
PI3K (p120g)(h)
PI3KC2a(h)
PI3KC2g(h)
Pim-1(h)
Pim-2(h)
Pim-3(h)
PIP4K2a(h)
PIP5K1a(h)
PIP5K1g(h)
MSC2360844
MSC2364588

F

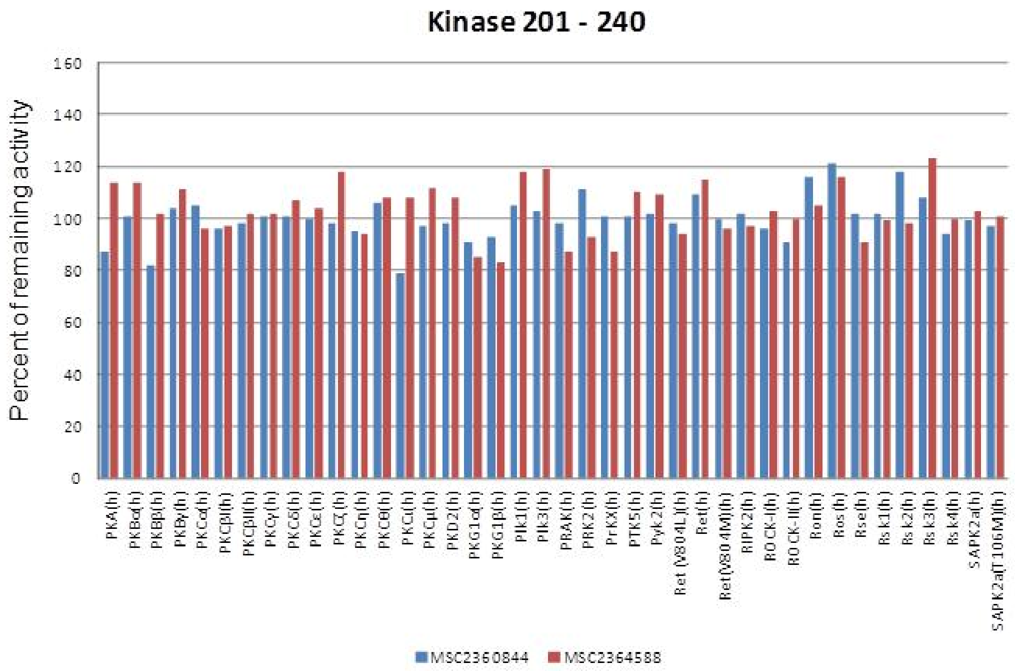
Kinase 201 - 240
Percent of remaining activity
0
20
40
60
80
100
120
140
160
PKA(h)
PKBα(h)
PKBβ(h)
PKBγ(h)
PKCα(h)
PKCβI(h)
PKCβII(h)
PKCγ(h)
PKCδ(h)
PKCε(h)
PKCζ(h)
PKCη(h)
PKCθ(h)
PKCι(h)
PKCμ(h)
PKD2(h)
PKG1α(h)
PKG1β(h)
Plk1(h)
Plk3(h)
PRAK(h)
PRK2(h)
PrKX(h)
PTK5(h)
Pyk2(h)
Ret (V804L)(h)
Ret(h)
Ret(V804M)(h)
RIPK2(h)
ROCK-I(h)
ROCK-II(h)
Ron(h)
Ros(h)
Rse(h)
Rsk1(h)
Rsk2(h)
Rsk3(h)
Rsk4(h)
SAPK2a(h)
SAPK2a(T106M)(h)
MSC2360844
MSC2364588

G

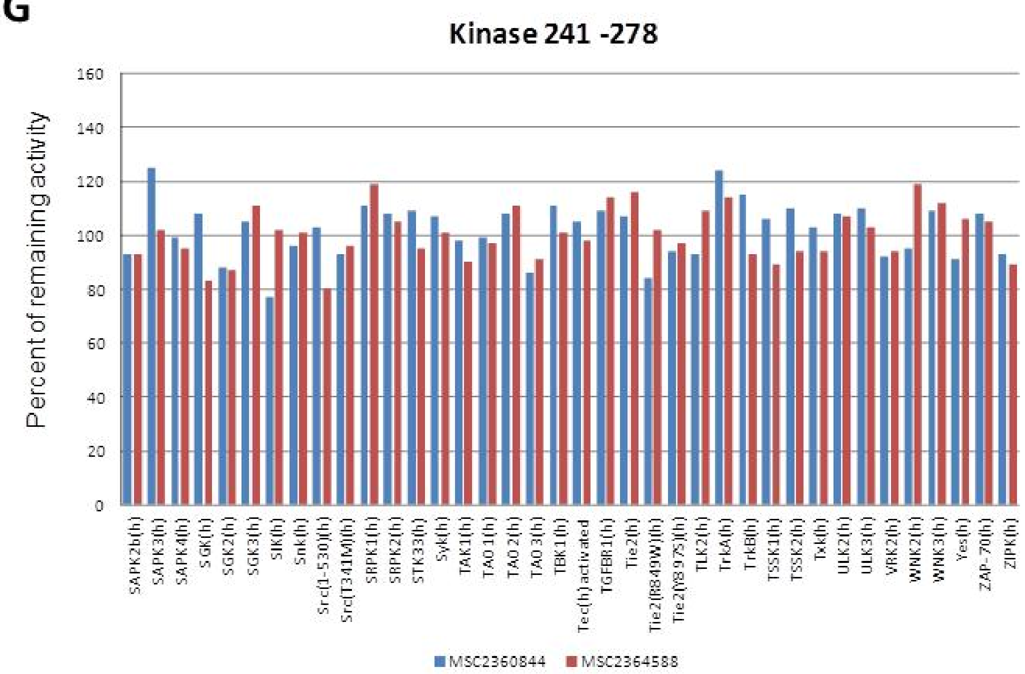

Supplemental figure 1

Kinase selectivity profile of MSC2360844 and MSC2364588 at 10 µM. Compounds were tested against 278 kinases, at the Km concentration for ATP for each kinase. Shown is a histogram representing the percentage of remaining activity compared to maximal activity in the absence of the compound.

2.2. Supplementary Figure 2. PK/PD correlation of PI3Kδ inhibitors in mice

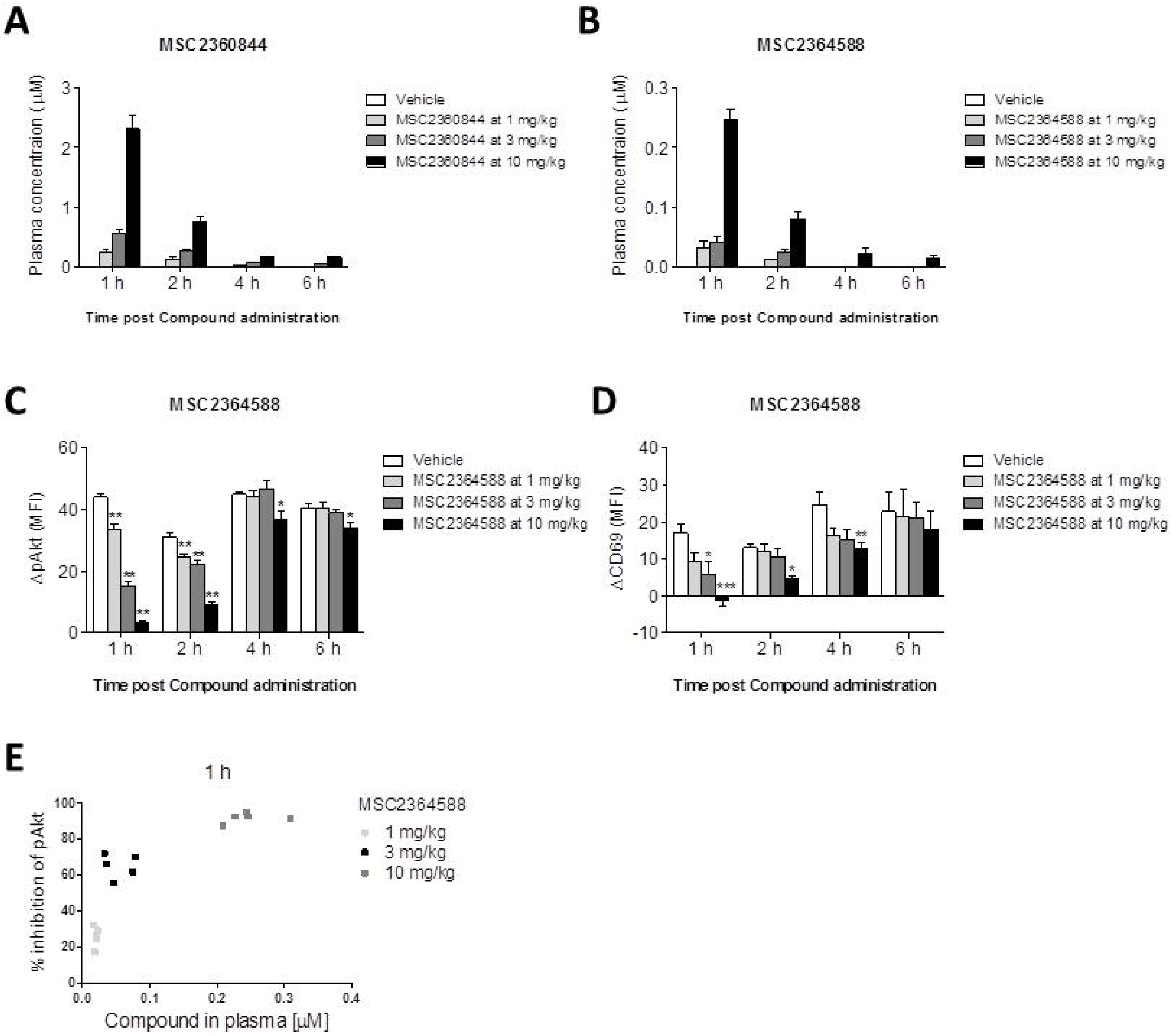

Supplementary Figure 2

PK/PD correlation of MSC2360844 and MSC2364588 in mouse. (**A-B**) *Ex vivo* assays. MSC2364588 at indicated doses was administered by gavage to naive mice at indicated time points before whole blood was withdrawn. In **A**, ΔpAkt was induced and measured as in Figure 5**C**. In **B**, CD69 was induced and measured in Figure 5**D**. Data shown are mean ± SEM with 5 mice per group and were analyzed by One way ANOVA followed by Dunnett's post-tests compared with vehicle group. *, $p < 0.05$; **, $p < 0.01$, ***, $p < 0.001$. (**C**) Plasma concentration of MSC2360844 after oral administration as in Figure 5C. Data are shown as mean ± SEM with 5 mice per group. (**D**) Plasma concentration of MSC2364588 after oral administration as in Supplemental Figure 2**A**. Data shown are mean ± SEM with 5 mice per group. (**E**) MSC2364588 concentrations in plasma were plotted against percentage of pAkt inhibition at 1h time point.

2.3. Supplementary Figure 3. Cytokines detected in kidney lysate at the end of experiment

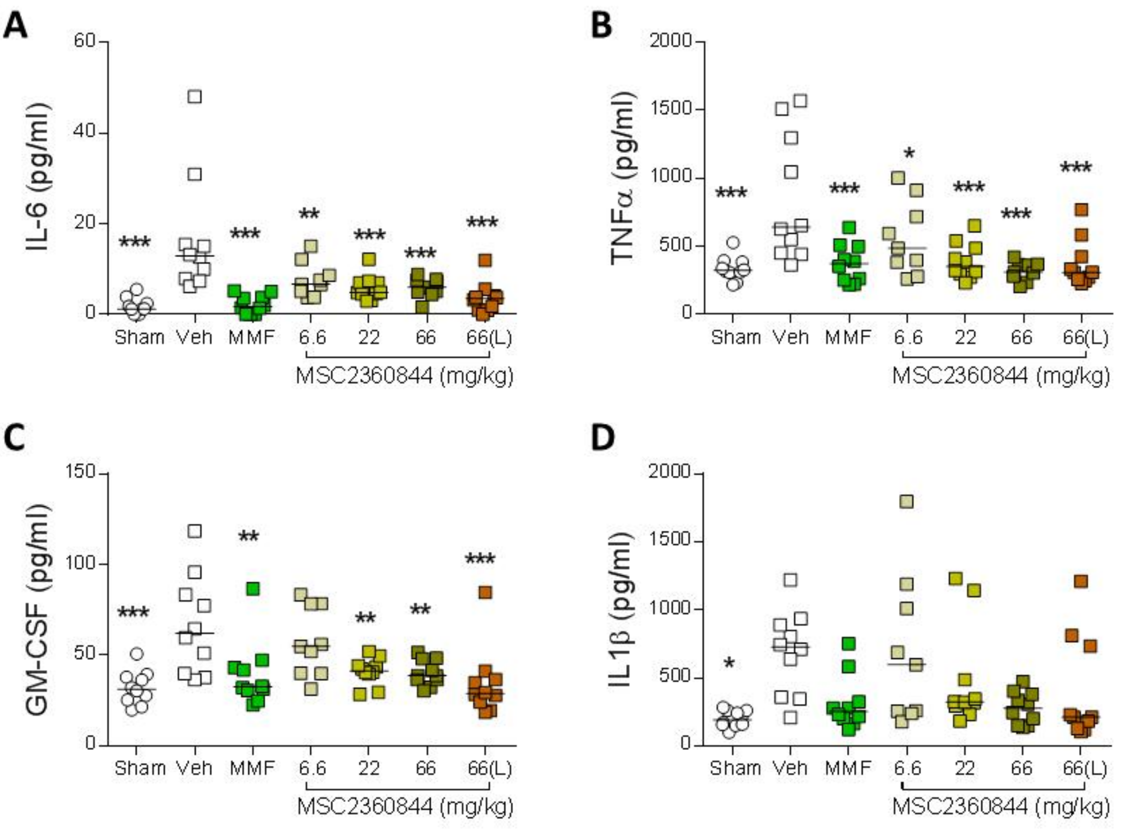

Supplementary Figure 3

MSC2360844 reduces cytokine levels in the kidney. Mice were treated as in Figure 6A. (**A**) IL-6, (**B**) TNFα, (**C**) GM-CSF and (**D**) IL-1β detected in the kidney lysates at the end of the experiment. For statistical analysis, One way Anova followed by Dunnett´s all groups compared to vehicle treated group was performed. *p< 0.05, **p< 0.01, ***p< 0.001

2.4. Supplementary Figure 4. Effects of MSC2364588 in IFNα-accelerated NZB/W F1 SLE model

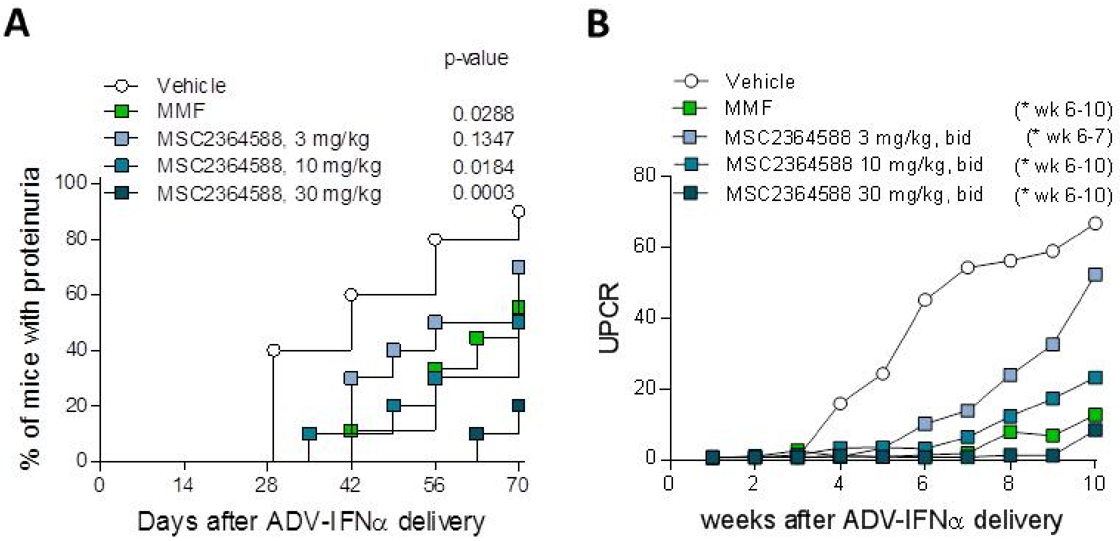

Supplementary Figure 4

MSC2364588 reduces disease severity in accelerated NZB/W F1 SLE model. (**A**) Incidence of proteinuria and (**B**) mean UPCR overtime in mice treated once daily with MSC2360844 at 6.6, 22 or 66 mg/kg, or MMF at 300 mg/kg with an early dosing regimen (starting at d14). For statistical analysis, in **A**, Log-rank (Mantel-Cox) method was used comparing all groups with vehicle group; in **B**, two-way ANOVA followed by Bonferroni post-test was used comparing all groups with vehicle group. Period of statistically significant differences are indicated with asterisk. *p< 0.05, **p< 0.01

2.5. Supplementary Figure 5. Time course of Ab secreting cells and cytokine –producing T cells in ADV-IFNα-accelerated NZB/W F1 SLE model

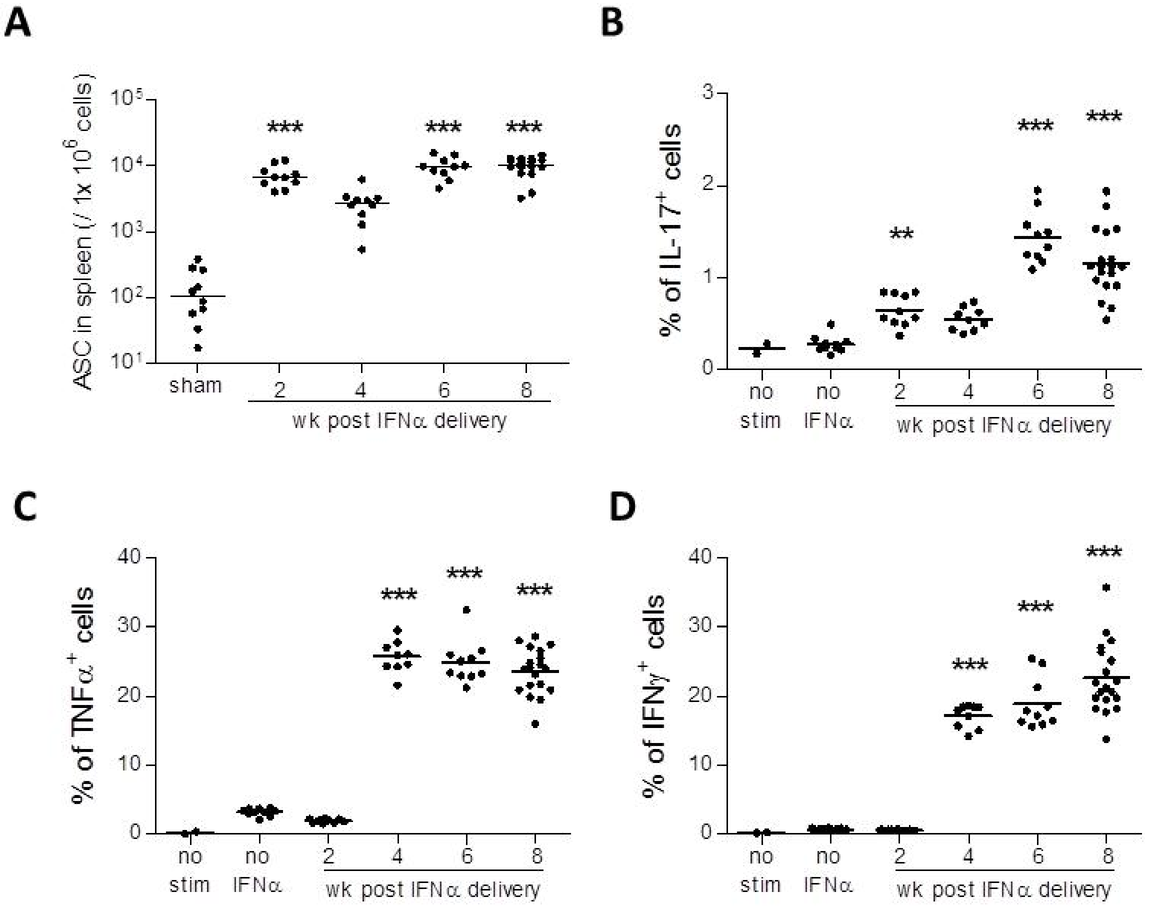

Supplementary Figure 5

Kinetics of spleen ASC and cytokine–producing T cell frequency in IFNα-accelarated NZB/W F1 SLE model. Spleen were taken from NZB/W F1 mice at different time points either with or without application of ADV-IFNα. ASCs were measured by ELISpot. Cytokine-producing T cells were measured by flow cytometry following intracellular staining. Each dot represents one mouse. Bar represents mean value. For statistical analysis, one way ANOVA followed by Dunnett´s was performed comparing all groups to the group without ADV-IFNα application. **p< 0.01, ***p< 0.001

3. Supplementary Methods: BioMAP® systems

3.1. Assay systems

Preparation and culture of endothelial cells and methods for the 3C, LPS, SAg and HPNo systems were as previously described [1;2]. PBMCs were prepared from buffy coats from normal human donors according to standard methods. For the HDF systems (/HDF3CT, HDF3CGF), human neonatal foreskin fibroblasts (HDFn) from 3 donors were pooled and cultured according to the supplier's (Lonza, Inc., Allendale, NJ) recommendation. HDFn were plated in low serum conditions 24 h before stimulation with cytokines. For the BE4T system, primary human bronchial epithelial

cells (Cell Applications, Inc., San Diego, CA), were cultured according to methods recommended by the manufacturer. Primary human keratinocytes were purchased from Lonza, Inc., Allendale,NJ). Positively selected primary normal human CD19+ B cells were obtained from AllCells (Emeryville, CA). Macrophages were derived in culture from normal peripheral blood CD14+ monocytes (AllCells, Emeryville, CA). Th2 cells were derived in culture from purified CD4+ T cells (AllCells, Emeryville, CA).

3.2. Compounds and reagents

Mouse antibodies against human biomarkers were obtained from commercial sources including BD Biosciences (San Jose, California), R&D Systems (Minneapolis, MN), Caltag/HBT/Invitrogen (Carlsbad, California), Sigma (St. Louis, MO), Fitzgerald Industries International, (Concord, MA), Abcam (Cambridge, MA), BioDesign International (Saco, Maine),Oncogene/EMD(Gibbstown, NJ), Neomarkers/Lab Vision (Fremont, CA), and Chemicon International/Millipore (Billerica, MA). Compounds were tested at various concentrations. Compounds were prepared in DMSO (final concentration 0.1–0.2%), added 1 h before stimulation of the cells, and were present during the entire 24 h (or 72 h) stimulation period. Two positive control (1.1 uM colchicine) and 8 negative control wells (DMSO) were employed on each plate.

3.3. Readout measurements

The levels of readout parameters were measured by ELISA [1;2]. For this procedure, supernatants from microtiter plates containing cell cultures are removed and plates are dried. Plates are then blocked by incubating with 0.01% non-fat dry milk in PBS containing 0.01% NP-40 for 1 h, and then incubated with primary antibodies or isotype control antibodies (0.01–0.5 µg/ml) diluted in PBS for 1 h. After washing, plates were incubated with a biotinconjugated anti-mouse IgG antibody (Cedarlane Laboratories, Burlington, NC) for 1 h followed by streptavidin-HRP (Jackson Labs, Bar Harbor, ME) for 30 min. Plates were washed in PBS and developed with TMB substrate (Clinical Science Products, Inc., Mansfield, MA) and the absorbance (OD) was read at 450 nm (subtracting the background absorbance at 650 nm). Quantitation of PGE2 and TNF-αin the LPS system was done using commercially available kits from Cayman Chemical (Ann Arbor, MI) and Invitrogen (Camarillo, CA), respectively, according to the manufacturer's directions. Overtly adverse effects of compounds on cells were determined by 1) measuring alterations in total protein (SRB assay), 2) measuring the viability of peripheral blood mononuclear cells. Proliferation of PBMC (T cells) was quantified by Alamar blue reduction and proliferation of adherent cell types was quantified by SRB staining. PBMC viability was assessed by adding Alamar blue (Invitrogen, Camarillo, CA) to PBMC that had been cultured for 24 h in the presence of activators and compounds and measuring its reduction after 8 h. SRB was performed by removing supernatants from plates, washing plates with PBS, then fixing cells with addition of 10% TCA for 1 h. Plates are washed again with PBS then, 0.1% sulforhodamine B in water is added and plates are incubated for 15min. Following wash with 1% acetic acid, plates are developed by the addition of 200µl per well of 10 mM Tris-base for 15 min and plates are read at 560 nm.

3.4. Statistical analyses

Statistical methods have been described previously [1;2]. Measurement values for each biomarker readout in a treated sample were divided by the mean value from 8 DMSO control samples (from the same plate) to generate a ratio. All ratios were then log10 transformed. Significance prediction

envelopes were calculated for historical controls (99% and 95%). Overtly cytotoxic compounds are identified as generating profiles with the log10 ratio of SRB levels<−0.3 in multiple systems. For analysis of profile similarities, overtly cytotoxic compound profiles were removed. In addition, in the present studies, the correlation metric was modified to include a combination of similarity metrics in addition to Pearson's correlation. This approach was found to improve the accuracy of mechanism classification with test data sets (not shown), due to the diversity of BioMAP profile characteristics (wide variation in the number of active readouts, the number of active systems, and in the amplitude of biomarker readout changes). Thus, the similarity metrics used for the analyses of profiles included Pearson's correlation, a real value Tanimoto metric (=A·B/ (||A||+||B||−A·B, where A and B are the two profile vectors), and a system weighted-averaged real value Tanimoto metric (=∑(i= system) Wi*Ti/∑Wi, where Ti is the real value Tanimoto score for the ith system, Wi is the weight for the ith system, Wi=number of markers in the system / (1+ exp(−(max. ratio of the two profiles in this system−0.09) * 100)). The real value Tanimoto metric was employed as a scaled version for filtering profile.

4. References[1]

[1] E.J. Kunkel, M. Dea, A. Ebens, E. Hytopoulos, J. Melrose, D. Nguyen, K.S. Ota, I. Plavec, Y. Wang, S.R. Watson, E.C. Butcher, E.L. Berg, An integrative biology approach for analysis of drug action in models of human vascular inflammation. FASEB J. 18 (2004) 1279-1281.

[2] E.J. Kunkel, I. Plavec, D. Nguyen, J. Melrose, E.S. Rosler, L.T. Kao, Y. Wang, E. Hytopoulos, A.C. Bishop, R. Bateman, K.M. Shokat, E.C. Butcher, E.L. Berg, Rapid structure-activity and selectivity analysis of kinase inhibitors by BioMAP analysis in complex human primary cell-based models. Assay.Drug Dev.Technol. 2 (2004) 431-441.

[1] Provide the doi when available, and ALL complete author names.
